# Differences among brain tumor stem cell types and fetal neural stem cells in focal regions of histone modifications and DNA methylation, broad regions of modifications, and bivalent promoters

**DOI:** 10.1186/1471-2164-15-724

**Published:** 2014-08-27

**Authors:** Sally Yoo, Mark C Bieda

**Affiliations:** Department of Biochemistry and Molecular Biology, University of Calgary, Calgary, Alberta Canada

**Keywords:** Cancer stem cells, Epigenetics, Chromatin immunoprecipitation, Histone modifications, Bivalent promoters

## Abstract

**Background:**

Aberrational epigenetic marks are believed to play a major role in establishing the abnormal features of cancer cells. Rational use and development of drugs aimed at epigenetic processes requires an understanding of the range, extent, and roles of epigenetic reprogramming in cancer cells. Using ChIP-chip and MeDIP-chip approaches, we localized well-established and prevalent epigenetic marks (H3K27me3, H3K4me3, H3K9me3, DNA methylation) on a genome scale in several lines of putative glioma stem cells (brain tumor stem cells, BTSCs) and, for comparison, normal human fetal neural stem cells (fNSCs).

**Results:**

We determined a substantial “core” set of promoters possessing each mark in every surveyed BTSC cell type, which largely overlapped the corresponding fNSC sets. However, there was substantial diversity among cell types in mark localization. We observed large differences among cell types in total number of H3K9me3+ positive promoters and peaks and in broad modifications (defined as >50 kb peak length) for H3K27me3 and, to a lesser extent, H3K9me3. We verified that a change in a broad modification affected gene expression of *CACNG7*. We detected large numbers of bivalent promoters, but most bivalent promoters did not display direct overlap of contrasting epigenetic marks, but rather occupied nearby regions of the proximal promoter. There were significant differences in the sets of promoters bearing bivalent marks in the different cell types and few consistent differences between fNSCs and BTSCs.

**Conclusions:**

Overall, our “core set” data establishes sets of potential therapeutic targets, but the diversity in sets of sites and broad modifications among cell types underscores the need to carefully consider BTSC subtype variation in epigenetic therapy. Our results point toward substantial differences among cell types in the activity of the production/maintenance systems for H3K9me3 and for broad regions of modification (H3K27me3 or H3K9me3). Finally, the unexpected diversity in bivalent promoter sets among these multipotent cells indicates that bivalent promoters may play complex roles in the overall biology of these cells. These results provide key information for forming the basis for future rational drug therapy aimed at epigenetic processes in these cells.

**Electronic supplementary material:**

The online version of this article (doi:10.1186/1471-2164-15-724) contains supplementary material, which is available to authorized users.

## Background

It is well-established that epigenetic marks are altered in cancer cells (e.g [1]). These alterations may play important roles in cancer [2, 3]. Different cancers appear to have different patterns of epigenetic changes. Importantly, drugs aimed at epigenetic processes are currently used for clinical treatment of cancer [3, 4]. Understanding the specific actions of these drugs and the genomic loci of their action may lead to future treatments that are more specific. Currently, there is intense work on developing new drugs aimed at epigenetic processes for cancer treatment and gaining a more detailed understanding of the specific steps involved in the action of these drugs on cancer cells. The efficacy of these drugs may rely, in part, upon the specific spectrum of epigenetic changes in the cancer cells and specific patterns of epigenetic changes may produce differential drug sensitivity among patients [3]. Hence, gaining a better understanding of epigenetic changes in different types of cancer cells is critical.

Glioblastoma multiforme is a deadly cancer under intensive investigation on a genomic level as part of the The Cancer Genome Atlas (TCGA) project [5]. In this cancer, it is believed that a specific population of cells - brain tumor stem cells (“BTSCs”) - plays a crucial role in the tumors. It has been proposed that targeting these cells may be essential for successful treatment of this cancer [6, 7].

Several previous findings point toward the need for systematic investigation of DNA methylation and the three histone marks H3K27me3, H3K9me3, and H3K4me3 in BTSCs. Changes in DNA methylation have been widely reported in cancer cells, are believed to play a major role in cancer cell behavior, and are the target of current drug and diagnostic marker development efforts [8]. Furthermore, changes in DNA methylation appear to be important in differentiation, including neural lineage cell differentiation [9]. Finally, in some BTSCs, the BMP differentiation pathway is silenced by aberrational methylation of the *BMPRB1* gene promoter, producing resistance to differentiation for these cells [10]. Hence, understanding DNA methylation patterns is critical. Similarly, H3K27me3 is important to investigate because it has repeatedly been shown to be an powerful repressive epigenetic mark in promoters, the underlying enzymes controlling this mark are known to be dysregulated or mutated in many cancers [11], and changes in the distribution of this mark are important in differentiation of neural stem cells [12]. In some BTSCs, EZH2, a key member of the PRC2 complex that mediates the production of H3K27me3 marks, is upregulated and plays a pivotal role in glioblastoma tumor growth [13]. H3K4me3 is important as a marker of open chromatin [14], has also been shown to change in localization during differentiation [15], and is strongly correlated with gene expression [16], and hence was also a target of this study. Finally, H3K9me3 is prevalent but shows a very divergent localization to H3K27me3 [16] and is well known as an important component of lengthy regions of heterochromatin in some cases [17]. In sum, these results strongly suggest pivotal roles for these epigenetic marks in BTSCs.

Here, we performed a preliminary survey of these epigenetic marks in four lines of brain tumor stem cells. These four lines have been shown to be multipotent [18]. We wished to compare our results to normal neural stem cells, which may be the cell type of origin for BTSCs [19]. To model this normal population, we used normal fetal human neural stem cells, which robustly grow under typical cell culture conditions. We employed chromatin immunoprecipitation applied to microarrays (ChIP-chip) and methylated DNA immunoprecipitation applied to microarrays (MeDIP-chip) technologies.

We investigated three fundamental manifestations [16, 20] of these four epigenetic marks. Best known are “focal peaks”, localized regions of epigenetic modification. Abundant evidence supports the key role of these modifications in transcriptional regulation when present in proximal promoters [21]. Less studied, but prevalent in the human genome, are long regions continuously possessing a epigenetic modification (“broad modifications”, [16, 22]). We operationally define a peak as a broad modification if it is >50 kb in extent. Many lengthy heterochromatic regions possess these long regions of H3K27me3 or H3K9me3 [16]. Finally, we also investigated bivalent promoters, which are promoters possessing two “opposing” epigenetic marks, usually H3K4me3 and H3K27me3 [20]. These bivalent promoters may play a key role in producing or maintaining fundamental stem cell properties [15]. Hence we investigated their distribution in BTSCs.

We addressed the following specific questions in this study. How similar are the BTSCs from an epigenetic mark perspective? How do BTSCs and neural stem cells differ in epigenetic mark localization across the genome? Do we observe differences in broad modifications among BTSC types and neural stem cells? How different are the collections of bivalent promoters among the BTSC types and fNSCs?

We began by determining a set of promoters that possess each mark in every BTSC type (“core set”). This core set was a substantial proportion of the total set of promoters bearing a given mark for each BTSC, indicating a significant core set but also significant epigenetic diversity among BTSCs. For each epigenetic mark, nearly all the core set was found in the corresponding fNSC set, indicating great commonalities between fNSCs and the common BTSC set of epigenetic mark localizations. However, fNSCs and BTSCs, when compared in a pairwise manner, revealed large differences. Therefore, although there is a substantial “core” set for each epigenetic mark, individual cell types have many other sites that are not shared with all the rest of the group. The total number of H3K9me3+ promoters varied markedly among some cell types, indicating differences in the activity of the H3K9me3 production/maintenance system among cell types. This system may present possibilities for therapeutic intervention in some groups of patients. We found that “broad modifications”, large areas of genomic space possessing an epigenetic mark (operationally defined as peaks >50 kb), also showed marked differences in presence or extent among cell types. We demonstrated that these differences were transcriptionally relevant. Given that the mechanisms producing these broad regions are significantly different than those producing focal peaks, this system may also present therapeutic possibilities. Finally, we surprisingly found that the collection of bivalent promoters also differs greatly by BTSC type, even though all our cell types were multipotent [18]. Only a small number of bivalent promoters were common to all five cell types. These observations suggest that bivalent promoter roles should be further investigated in these cells. In total, our results provide first steps toward understanding the epigenome of these cells and indicate that successful targeting of BTSCs using epigenome-aimed drugs may require careful patient-by-patient analyses.

## Results

### ChIP-chip and MeDIP-chip in BTSCs and fNSCs

To investigate the distribution of epigenetic marks, we used the 2.1 million probe NimbleGen HD2 microarray, a design centered around promoter regions, most of which are 10 kb in extent with 7 kb upstream and 3 kb downstream of the transcription start site and 100 bp probe spacing. In addition, this array design also includes a set of large tiled regions (the 44 ENCODE regions; for full array description, see Methods). We performed typical ChIP-chip and MeDIP-chip protocols (see Methods). We produced single microarray experiments for our twenty conditions (five cell types x four epigenetic marks).

We used several approaches to validate our microarray data. We started with the classical approach of examining known sites of modification (positive control sites) in our microarray data (Additional file 1: Figures S1-S5). Examination of this data for each mark revealed not just consistent presence of the expected epigenetic mark, but also that the waveforms were very similar across different cell types. This suggests that technical noise was quite low. In addition, we compared our data to public ENCODE consortium data for these same regions [21]. This ENCODE ChIP-seq data and methylation data from other cell types also closely matched the waveforms and location of our signals. This is consistent with reliable signals across experiments.

We next sought to examine this issue on a global scale. We examined promoters as to enrichment for different marks by using the maxfour methodology (see Methods). These analyses have been previously used [23, 24]. Examples are displayed in Additional file 1: Figure S6. To begin, we expect that when a promoter in one cell type has a strong signal for H3K27me3, then that promoter will most often display a strong signal in a second, closely related cell type. Due to cell type differences, there will be some promoters violating this pattern. In Additional file 1: Figure S6A, we demonstrate that the enrichment values for H3K27me3 are well-correlated between B73 cells and fNSCs, with some clear sites showing cell-type specificity. In contrast, as expected, few promoters have both high H3K9me3 and H3K27me3 values (Additional file 1: Figure S6B). Similarly, few promoters have both high H3K4me3 and H3K27me3 signals (Additional file 1: Figure S6C). These graphs closely resemble previous published results and closely match expectations from previous studies [23, 24]. Finally, we used the following logic. In a set of distinct, but closely related cell types, we expect that many positive sites that show variability between cell types will still be found in at least two cell types. We found that the great majority of promoters showing a signal for an epigenetic mark (“positive promoters” for that mark; described in detail below) were found in at least two cell types and a large number were found in all four BTSC types (Additional file 1: Figure S7A). In contrast, we found that promoters positive in only one cell type were rare, except for the case of H3K9me3 (discussed below) (Additional file 1: Figure S7B).

### Similar numbers of sites across cell types except in the case of H3K9me3

We examined differences among cell types using two different approaches for each epigenetic mark: total number of positive sites (“peaks”) and total number of proximal promoters possessing at least one peak (“positive promoters”). To determine the number of positive sites (“peaks”), we used the full set of peaks supplied by NimbleGen (“generous parameters”; see Methods). We focused on proximal promoter regions because these regions are key for epigenetic transcriptional regulation. Also, these regions are well-represented in this microarray design. To study these regions, we took positive sites (“peaks”) and mapped them to an existing Nimblegen proximal promoter array design in which the tiled region for promoters is designed to span the transcription start site (typically 3.5 kb upstream and 750 bp downstream; see METHODS). Then, we could score each promoter as positive for a given mark or negative based on presence of a peak in this proximal promoter. All analyses in our work used this promoter-centric dataset unless otherwise noted. We also repeated our basic analyses (e.g. generation of positive promoters for each cell type) using a more stringent peak selection criterion (FDR < 0.2; see METHODS for further details and data files).

**Figure 1:**
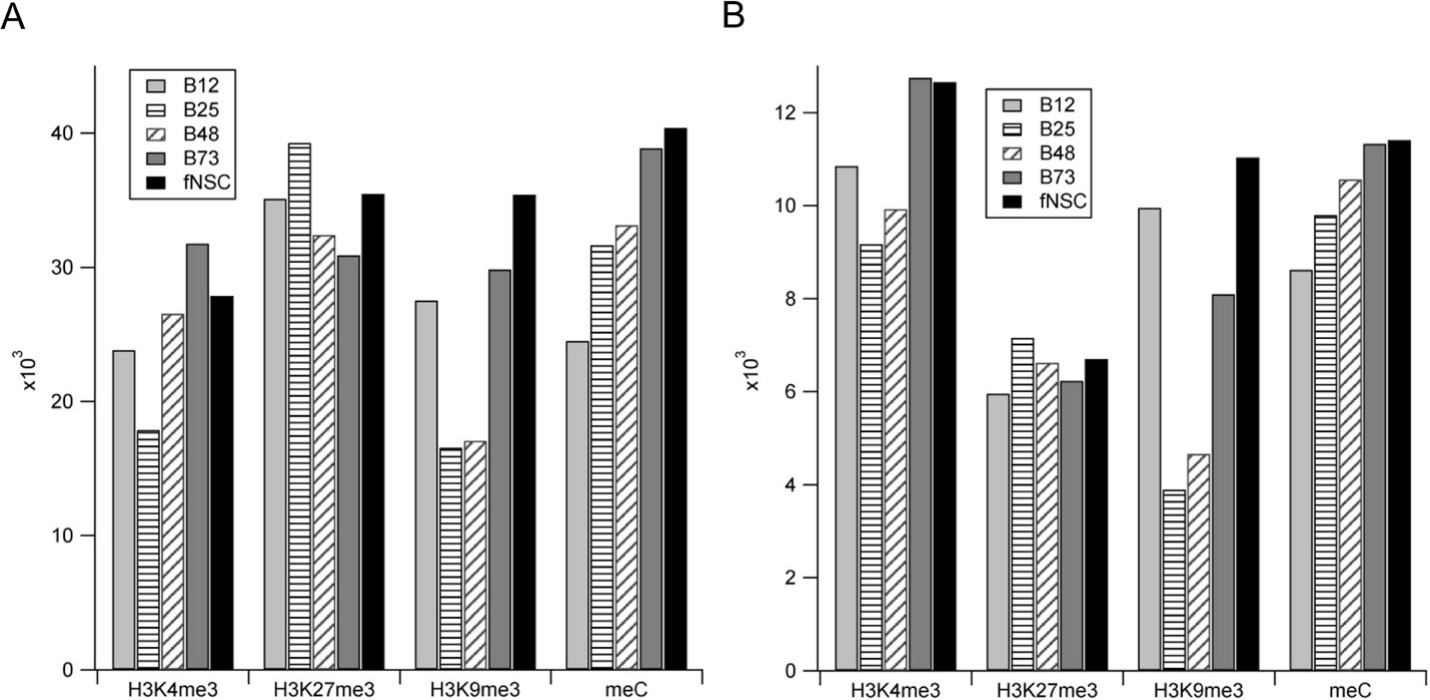
**Number of peaks and peak-containing promoters for each cell type and epigenetic mark. A**. Number of peaks found per epigenetic mark per cell type. This analysis used generous parameters leading to relatively large numbers of potential peaks. **B**. Number of promoters possessing each epigenetic mark. Peaks were mapped to proximal promoter regions; if a peak (or multiple peaks) overlaps a promoter, the promoter was scored as positive (see RESULTS and METHODS for details). Note large differences among cell types in number of H3K9me3+ promoters.

The results of these analyses are displayed in Figure 1. These analyses revealed one striking result and one more minor observation. For H3K9me3, there were large differences in number of sites and number of positive promoters across cell types, with the B12 line and fNSCs having many more positive promoters than B25 cells (>2× difference). Second, a more minor observation concerns the relative number of H3K4me3 and H3K27me3 peaks vs positive promoters. There were more H3K4me3 positive promoters than H3K27me3 positive promoters, as opposed to the total peak analysis. This result implies that a large proportion of H3K27me3 peaks were found outside of proximal promoter regions or that there was a greater number of H3K27me3 peaks per promoter.

**Figure 2:**
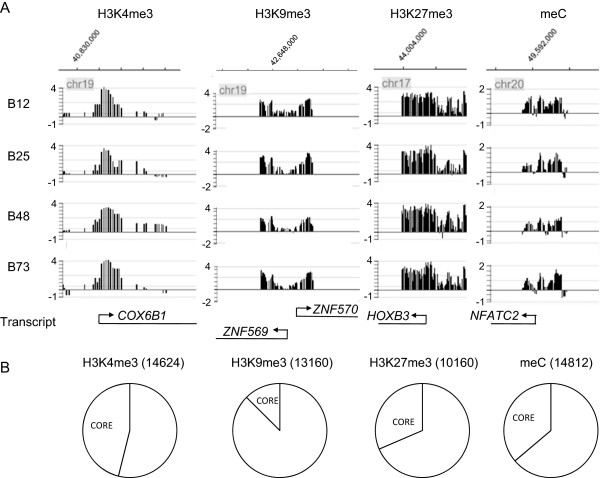
**Universe of promoters that possess each epigenetic mark. A**. Examples of each epigenetic mark for a single promoter from each BTSC type. Note great similarity in waveforms across cell types for a given epigenetic mark. Left axis is log2 enrichment ratio. For genomic location (top axis), only single coordinate is shown; genomic distance between tick marks is (panels from left to right): 1 kb, 6 kb, 4 kb, 8 kb. All displayed transcripts extend beyond edge of figure area. **B**. Diagram of number of promoters that possess a given epigenetic mark in at least one type of BTSC (“universe” set; number in parentheses) and the number that possess it in every BTSC type (“core” set). Number in core set: H3K4me3: 6742; H3K9me3: 1650; H3K27me3: 3198; meC: 5360. See RESULTS and METHODS for details of computation of sets.

We found the H3K9me3 difference between B25 and B12 cells surprising because our close examination of some known sites (Figure 2, Additional file 1: Figures S3,S4) did not demonstrate any obvious differences. The B25 peak waveforms and amplitudes at these control sites did not differ from those of the other cell types in this study. Therefore, we employed two additional global analysis approaches. First, analysis of only high confidence peaks (FDR < 0.2; see Methods for additional description) also yielded large differences between B12 cells and B25 cells in both total number of peaks and total number of positive promoters for H3K9me3 (Additional file 1: Figure S8). Hence, the observed differences cannot be attributed to the use of generous parameters. Second, we used the maxfour approach (maxfour; see [23] and Methods) beginning with quantile normalized and smoothed data to examine this conclusion. This method predicted the same rank order as the NimbleGen analyses in number of positive promoters (i.e. fNSC > B12 > B73 > B48 > B25). Furthermore, examination of the distribution of maxfour values revealed a strong positive shift in the B12 distribution as compared to that of B25 cells, indicating that many more promoters had higher H3K9me3 signals in B12 cells (data not shown). Therefore, use of two different analysis approaches using two different data preprocessing methods (none for NimbleGen, quantile normalization + smoothing for maxfour), and varying parameters for the NimbleGen peak determination, yielded qualitatively similar conclusions. This large difference in H3K9me3 positive promoter counts among these cells points toward a significantly altered H3K9me3 system for some types of BTSCs as compared to fNSCs.

### Similarities and Differences Among BTSCs in Epigenetic Mark Locations

Epigenetic marks are controllers of gene transcription and hence play an essential role in establishing cellular identity. Different types of cells can be distinguished by comparing epigenetic mark distributions and these distributions can even be used to predict relatedness of cell types [25]. We sought to address the issue of epigenetic similarities and differences among BTSCs. We used the promoter-centric analysis because marks in proximal promoter sites are crucial for transcriptional control. Furthermore, this analysis approach eliminates some methodological noise concerns (see Methods). Additionally, we validated qualitative results using a different analysis method (rank analysis as in [25]; data not shown). For each epigenetic mark, we computed two different basic sets. First, we computed a “core” set of positive promoters for each mark that are present in every BTSC (Additional files 2, 3, 4 and 5). These core sets probably contain epigenetic modification sites that produce the BTSC phenotype. Second, to gain an idea of the total variability in locations among cell types for a given epigenetic mark, we also computed a “universe” set that includes every promoter that is positive for a given epigenetic mark, whether in only one type of BTSC, or in two, three, or all four types (Additional files 6, 7, 8, and 9).

To interpret this data, we employed the following logic for each epigenetic mark. If different BTSCs share most of their positive promoters, then the core set will be a large fraction of the universe set. Conversely, if there are fewer shared positive promoters, then the core set will be a small fraction of the universe set, because most positive promoters will not be present in all four cell types.

Analysis of this data yielded several conclusions. First, there was a substantial core set of positive promoters for each epigenetic mark (H3K4me3: 6742; H3K9me3: 1650; H3K27me3: 3198; meC: 5360). Second, this core set, although large, was less than half of the universe set in each case (Figure 2). We were concerned that the relative size of the core set would be greatly reduced if there were a single “outlier” cell type that shared few sites with the other cell types. To investigate this possibility, we performed a more detailed analysis categorizing each site as belonging to either exactly one, two, three or all four types of BTSCs (Additional file 1: Figure S7). These results, in conjunction with detailed analysis of the composition of sites found in the “only present in one type” set, indicated that no single cell type was strongly biasing results. Overall, these results establish a large core set for each epigenetic mark that will be useful for further functional understanding of the general features of the BTSC phenotype. However, these results also demonstrate significant differences in the epigenetic landscape among BTSCs.

We performed gene ontology analysis using the DAVID server [26] for each of the core sets (full results in Additional files 10, 11, 12, and 13). For H3K4me3, we found enrichment of a large number of categories, with prominent enrichment in general gene ontology terms (e.g. “cytosol”). Notably, other terms potentially related to cancer also showed enrichment such as “GO: 0006974 ~ response to DNA damage stimulus” and “GO: 0007049 ~ cell cycle”. For H3K9me3, we found prominent enrichment of the cell adhesion and zinc-finger gene categories (INTERPRO Cadherin term, zinc finger regions (UP_SEQ_FEATUREs), and consistent gene ontology categories for these (GO: 0007156 ~ homophilic cell adhesion, GO: 0003677 ~ DNA binding)). For H3K27me3, analysis revealed enrichment of categories related to cell surface receptors, in particular G-protein coupled receptors and ion channels. Finally, for methylated DNA, analysis showed marked enrichment in categories related to transcriptional regulation and DNA binding categories. In sum, these results imply a complicated scenario in which there is potentially prominent and pan-BTSC repression of various transcriptional regulation genes (via H3K9me3 or DNA methylation) and cell surface molecules (some receptors via H3K27me3+ and some cell adhesion genes via H3K9me3), along with activation of various genes potentially linked to the cancer phenotype (via H3K4me3; e.g. cell cycle genes). Further exploration of these topics will probably require extensive experimentation, including large scale gene expression data.

### Epigenetic differences among fNSCs and BTSCs

There is significant evidence that neural stem cells may be the cell-type of origin for BTSCs [6, 7, 19]. We sought to determine changes in epigenetic mark locations induced by the putative transformation of neural stem cells (which we modeled with fNSCs) to BTSCs. We reasoned that these epigenetic alterations may give insight into (1) key sites of epigenetic marks that produce or contribute to the cancerous BTSC phenotype or (2) may provide avenues for therapeutic intervention, even if these new sites do not directly produce the cancer phenotype. These analyses rely on the assumption that human fNSCs are a good model for the epigenetic marks of the normal cell-type of origin for our BTSCs.

**Figure 3:**
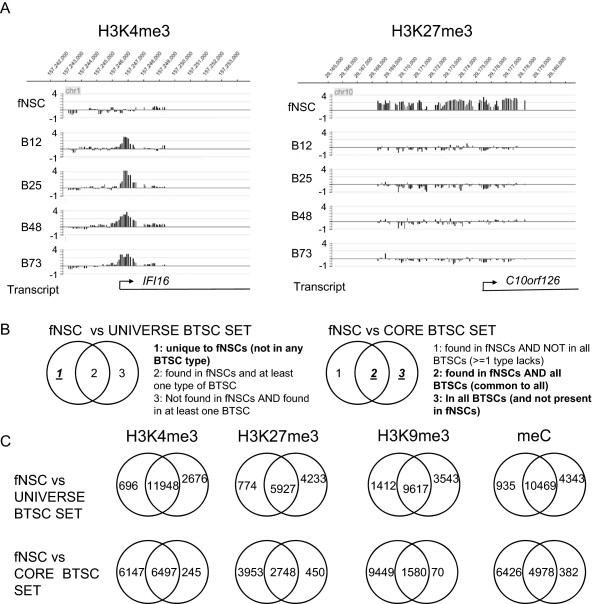
**Comparison of fNSCs and BTSCs.** Human fetal neural stem cells were used as model for the normal precursor cells for BTSCs. **A**. (left) Example of gain of H3K4me3 mark in a promoter in all four BTSC lines, as compared to fNSCs. (right) Example of loss of H3K27me3 mark in all four BTSC lines, as compared to fNSCs. Left axis is log2 enrichment ratio. All displayed transcripts extend beyond edge of figure area. **B**. Explication of comparison of fNSC promoter sets to BTSC promoter sets by epigenetic mark. “Universe” and “core” sets as in Figure 2. fNSC is left circle in each case. Critical computations in diagram are indicated in bold, italic, underlined font. **C**. Results of comparison of fNSC and universe or core BTSC sets for each epigenetic mark.

We are interested in two basic set of changes (Figure 3). First, we examine which epigenetic marks at which promoters are changed in *every* BTSC as compared to fNSCs. This conservative set may point toward key sites and potentially provide mechanistic insights into this reprogramming. Second, we examine which epigenetic marks are changed in *at least one type* of BTSC as compared to fNSCs. This larger set may point toward a set of promoters than *can* be epigenetically reprogrammed in the cancer process and, via overall analysis, may give insight into underlying mechanisms or molecular components of this large-scale reprogramming. On a technical level, this set is also important for derivation of changes that occur in comparing fNSCs to every BTSC (see below and Figure 3).

We were particularly interested in alterations found in every BTSC vs fNSCs. Full datasets are present in Additional files 14, 15, 16, 17, 18, 19, 20, 21, 22, 23, 24 and 25. As shown in Figure 3A, these changes could be gain of an epigenetic mark in every type of BTSC (Figure 3Aleft) or loss of an epigenetic mark in every type of BTSC (Figure 3Aright). To derive these changes, we examined differences among the fNSC set and the universe and core sets of BTSC promoters for each epigenetic mark (logic is displayed in Figure 3B).

This analysis revealed a relatively small set of changes that occurred in every BTSC vs. fNSCs (Figure 3C). For H3K4me3, H3K27me3 and meC, the number of promoters that lost the marks (in the putative neural stem cell - > BTSC process) significantly outweighed the number of promoters that gained the marks (quantitation in Figure 3C). For H3K9me3, the difference was very large (1412 lost vs 70 gained). The large number of H3K9me3 locations that are lost and the small number gained may be due to the small size of the core H3K9me3+ promoter set (Figure 2B). The small core set size is partially caused by large differences among BTSC types in number of H3K9me3 marked promoters, as analyzed above (Figure 1, Additional file 1: Figure S7).

Inspection of the universe set results indicated that, overall, thousands of promoters can be reprogrammed in the putative NSC - > BTSC transformation for each epigenetic mark.

We examined the composition of the group of consistent precursor cell (fNSC as model) to BTSC changes (changed in every BTSC cell) for each epigenetic mark using the DAVID server (full data in Additional files 26, 27, 28, 29, 30, 31, 32 and 33). We separately analyzed sets of gains and losses. Gene ontology analysis revealed only a few notable upregulated categories. For H3K9me3, BTSCs gained this mark for zinc finger genes. For H3K27me3, BTSCs gained this mark for receptors, especially G-protein coupled receptors, and lost this mark in the categories of embryonic limb morphogenesis and skeletal development. For DNA methylation, there were clear gains in genes related to the general categories of transcription and development and clear losses in genes in the categories of receptors and the general category of membrane. Overall, these results point toward a complex picture in which potentially some zinc finger genes gain repression (via gain of H3K9me3), some receptors are newly repressed (via gain of H3K27me3+) and other receptors are potentially activated (via loss of DNA methylation), and genes related to transcription are also potentially repressed (via gain of DNA methylation) and some developmental genes are activated (via loss of H3K27me3+) and some repressed (via gain of DNA methylation). The core data sets themselves have a biased gene ontology category composition, so these results are in part due to core set composition effects. Further experimental investigation of BTSCs will be required to address the specifics of these processes.

### Regions of Broad Modification Vary Among Cell Types

We use the term “broad modification” to denote long regions of chromosomal space in which there is a given epigenetic modification present continuously (>50 kb), as opposed to focal regions of modification (“peaks”) in a promoter or other region. These broad modifications are prevalent in the human genome [16]. The mechanisms that create broad modifications are believed to be significantly different from those that create focal modifications [22]. Hence, variations among cell types in this type of modification implicates mechanisms different than those involved in focal peak dynamics. Here, we examine variation in broad modifications among our five cell types.

**Figure 4:**
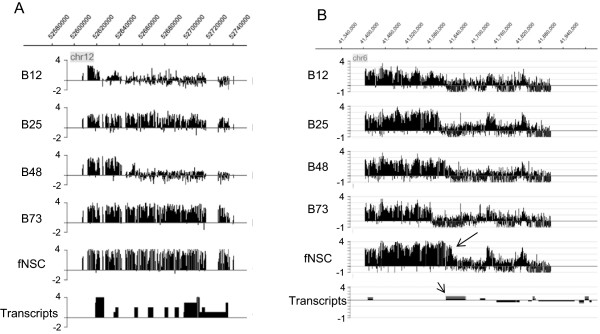
**Examples of variation among cell types in the extent of broad regions of H3K27me3. A**. HOXC cluster region of chromosome 12. Note the large differences between B73 and B12 in particular. Left axis is log2 enrichment ratio. **B**. Region of chromosome 6. Large arrow indicates differential region, which encompasses transcription start site for *FOXP4* gene (gene indicated by smaller arrow in Transcripts track). Note particularly large difference between B73 and fNSC. Markings for “Transcripts” tracks: boxes above the line indicate transcripts that are on the + strand (i.e. TSS (transcription start site) on left side of box), while boxes below the line represent transcripts that are from the opposite strand (i.e. TSS is on right of box).

To study alterations in broad modifications, we examined the longer regions represented in this microarray format (see Methods; Additional file 34). Strikingly, cell type differences were found in 26 of 44 regions for H3K27me3 (e.g. Figure 4). In contrast, clear changes in broad modifications were much less prevalent for H3K9me3 (4 of 44) and were absent for meC and H3K4me3 (0 of 44 in each case). These results imply frequent dysregulation of H3K27me3 spreading and less prevalent changes in H3K9me3 spreading. Due to the limitations of this microarray format with respect to investigating broad modifications, conclusions around these results must be tentative.

**Figure 5:**
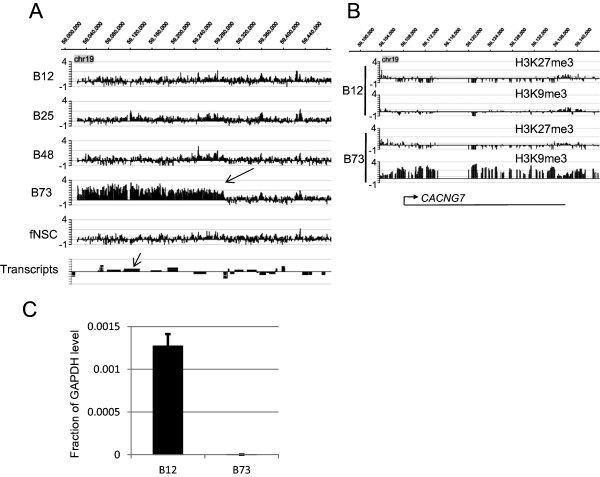
**Differential presence of a broad region of H3K9me3 affects transcription. A**. H3K9me3 ChIP-chip results. B73 cells show broad region of H3K9me3 that is missing in other cell types (large arrow). Small arrow (Transcripts track) indicates *CACNG7* transcript. **B**. Detailed view of this *CACNG7* region reveals H3K9me3 presence in B73 cells but not B12 cells. H3K27me3 is not present in either B73 or B12 cells in this region. **C**. Quantitative PCR of mRNA abundance (cDNA) demonstrates that B12 cells contain much higher levels of *CACNG7* than B73 cells. Values (mean + standard deviation) are expressed as fraction of the GAPDH level; t-test revealed p < 0.005 for this difference. Similar values were seen using ACTB as a control (see RESULTS).

We next examined whether broad region variation among cell types was relevant to transcriptional regulation. The chr19: 59023585 - 60024460 region encompasses the *CACNG7* gene (Figure 5A). This gene has been recently described as being routinely downregulated in BTSCs as compared to normal neural stem cells [27]. In this region, we found a broad modification (H3K9me3+) in B73 cells but not in B12 cells. This broad modification was restricted to H3K9me3 and was not present with H3K27me3 in this region (Figure 5B). We examined mRNA levels of *CACNG7* using quantitative PCR and found that B12 cells had higher levels than B73 cells (Figure 5C). This difference was very large (>1500 fold using either GAPDH or ACTB as a control transcript). Hence, the change in the presence of this broad region of H3K9me3 is correlated with a large difference in mRNA levels for *CACNG7*, suggesting that these changes in broad modifications can have transcriptional, and presumably ultimately functional, consequences.

### Bivalent Promoters

“Bivalent promoters” are promoters that possess two “opposing” epigenetic marks, most commonly H3K4me3 in combination with H3K27me3 [28]. This class of promoters is prominent in stem cells and is believed to contribute significantly to the multipotent nature of tissue-specific stem cells [29]. We surveyed bivalent promoters in BTSCs and sought to determine whether there were regular patterns of differences among fNSCs and BTSCs. As in previous analyses, we assume that the fNSC epigenetic patterns are a good model for those of the normal precursor cells of BTSCs.

**Figure 6:**
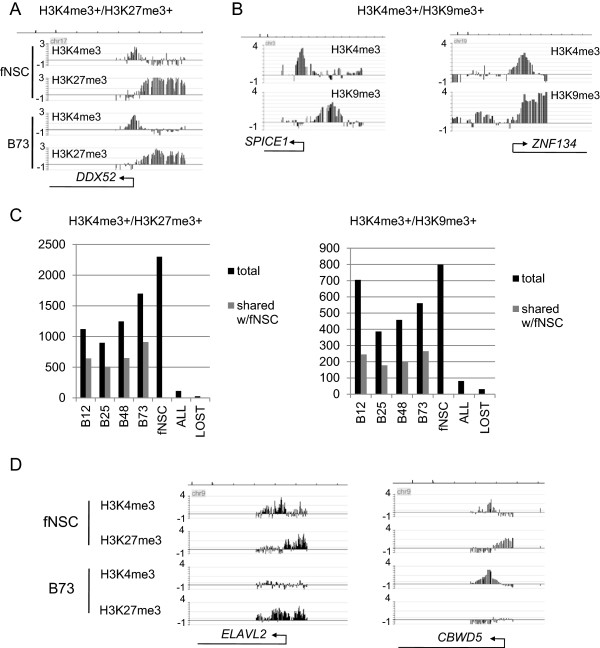
**Bivalent Promoters in fNSCs and BTSCs. A**. H3K4me3+/H3K27me3+ promoter in fNSCs and B73 cells. Note that both marks are in the proximal promoter but show little overlap. This was the most common pattern. Left axes are log2 enrichment ratio. **B**. Two patterns for H3K4me3+/H3K9me3+ promoters (B12 cells). (left) Non-overlapping marks in proximal promoter. This is the most common pattern. (right) Overlapping marks. Left axes are log2 enrichment ratio. **C**. (left) H3K4me3+/H3K27me3+ promoter counts in each cell line and the number shared with fNSCs. (right) H3K4me3+/H3K9me3+ promoters in each cell line and number shared with fNSCs. ALL refers to bivalent promoters found in all 5 cell types. LOST refers to promoters that are bivalent in fNSCs and lack both marks in every BTSC line. **D**. Examples of fNSC H3K4me3+/H3K27me3+ promoters that resolve to either H3K27me3+ (left) or H3K4me3+ (right) promoters in the B73 line. Left axes are log2 enrichment ratio. Markings: For **A**, **B**, and **D**, transcripts are shown with arrows and line drawing. All displayed transcripts extend beyond edges of panel in figure. Markings for top axes (genomic coordinate of first tick mark (increment to next tick mark)): **A**: chr17:33,063,000 (3 kb); **B**: left: chr3:114,711,000 (1 kb); right: chr19:62,812,000 (1 kb); **D**: left: chr9:23,790,000 (6 kb); right: chr9:68,541,000 (3 kb).

We used a conservative selection strategy to find proximal promoters that showed clear evidence of strong presence of two epigenetic marks (H3K4me3+/H3K27me3+ (“K4/K27 bivalent promoters”) or H3K4me3+/H3K9me3+ (“K4/K9 bivalent promoters”); see Methods for details) in a single promoter. Detailed lists are presented in Additional files 35, 36, 37, 38, 39, 40, 41, 42, 43 and 44. Visual inspection of a random subset of these bivalent promoters showed that, qualitatively, in the majority of cases, the epigenetic marks seemed to occupy different regions of the promoter (Figure 6A, B(left)). Hence, direct, clear overlap of marks for the same probes was observed infrequently (Figure 6B(right)). To quantify this observation, we performed a preliminary analysis of bivalent promoters in B73 cells. We analyzed each K4/K27 or K4/K9 promoter to determine if the peaks were overlapping or separate. We found that 37% (399 of 1083 analyzed) K4/K27 promoters and 40% (147 of 368 analyzed) of K4/K9 promoters had non-overlapping peaks (see Methods for analysis). Our analysis method will underestimate the fraction of non-overlapping histone modifications because peak widths are larger than the actual underlying chromatin modification. This is due to the size of chromatin fragments used in ChIP experiments. This situation produces apparent overlap when the underlying modifications are separated in genomic space. Nonetheless, these results support the existence of a major population of bivalent promoters with non-overlapping histone modifications. Modeling the relationship of underlying modification to peak morphology may allow better quantification in the future.

We began by analyzing K4/K27 bivalent promoters. We found that the number varied considerably among cell lines ranging from 898 in B25 cells to 2301 in fNSCs (Figure 6C). Analysis of bivalent promoter changes from fNSCs to any single type of BTSC demonstrated large alterations and substantial diversity among patterns in different cell types. There were few bivalent promoters found in all 5 cell types (5% of fNSC total; 113/2301) and fewer that went from bivalent in fNSCs to losing both marks in all BTSCs (going to K4-/K27- status) (1%; 26/2301). These results point toward notable diversity in K4/K27 bivalent promoters for each cell type.

We performed similar analyses with bivalent K4/K9 promoters. We found that the number of K4/K9 bivalent promoters also varied substantially among cell lines (Figure 6C(right)). Comparison of the fNSC set to sets from individual BTSC types revealed substantial overlap. However, there was only a small set of bivalent promoters found in all BTSCs and fNSCs, and fewer still that lost both marks in all BTSCs (going to K4-/K9-) as compared to fNSCs (Figure 6C). These results indicate considerable differences between each cell type in the set of K4/K9 bivalent promoters.

We were particularly interested in epigenetic differences between fNSCs and BTSCs at these bivalent promoters, because loss of bivalency is often considered an important step in differentiation. Alterations from K4+/K27+ to only K4+ would poise a promoter for activation (Figure 6D(right)). We found 137 promoters that went from K4+/K27+ in fNSCs to only K4+ in all BTSCs. Gene ontology analysis of the associated genes yielded mostly general categories except for categories linked to transcription (GO:0006355 ~ regulation of transcription, DNA-dependent) and proto-oncogenes (SP_PIR_KEYWORDS “Proto-oncogene” category).

In contrast, alterations from K4+/K27+ to solely K27+ would place a promoter in a repressive state (Figure 6D(left)). We found 191 promoters that went from K4+/K27+ in fNSCs to only K27+ in all BTSCs. Gene ontology analysis of the associated genes yielded categories (SP_PIR_KEYWORDS) linked to ion channels (“ionic channel”, “ion transport”, “voltage-gated channel”), but no statistically significant gene ontology categories.

Similar logic holds for K4+/K9+ promoters. We found 99 promoters that went from K4+/K9+ in fNSCs to only K4+ in all BTSCs. Gene ontology analysis of the associated genes yielded categories (SP_PIR_KEYWORDS) linked to transcription (“transcription”, “transcription regulation”) and “mental retardation”, but no statistically significant gene ontology consortium categories.

We found 22 promoters that went from K4+/K9+ in fNSCs to only K9+ in all BTSCs. Gene ontology analysis of the associated genes yielded a number of categories linked to cell adhesion, including GO:0016337 ~ cell-cell adhesion.

Taken together, these analyses point toward potential activation of genes linked to transcription and proto-oncogenes due to loss of the repressive mark (i.e. loss of either H3K27me3+ or H3K9me3+) and potential repression of genes linked to cell adhesion (K4+/K9+ to K9+) and ion channels (K4+/K27+ to K27+). Again, these results are in part related to the composition of the core sets for each epigenetic mark (see above analysis of DAVID results for core sets).

Full DAVID results are presented in Additional files 45, 46, 47, 48, 49, 50 and 51. Other bivalent promoter analyses are presented in Additional files 52, 53, 54, 55, 56 and 57.

## Discussion

In this report, we surveyed four well-studied epigenetic marks using ChIP-chip/MeDIP-chip in four brain tumor cell lines and fetal neural stem cells. We used fetal neural stem cells as a model for the normal cell type of origin of the brain tumor stem cells. The four epigenetic marks (H3K4me3, H3K27me3, H3K9me, DNA methylation) have been shown to play important roles in transcriptional regulation and function of neural precursor cells [30], the putative precursor cells for BTSCs [7, 19]. Our primary goals were to identify similarities and differences among the brain tumor stem cell lines and also to understand potential epigenetic reprogramming from neural stem cells to brain tumor stem cells. We surveyed various epigenetic phenomena, including focal peaks, areas of broad modification, and bivalent promoters. For most of our analyses, we performed a promoter-centric analysis by mapping peaks to proximal promoter regions, where the presence of an epigenetic mark has a great probability of affecting transcription [31]. Our primary conclusions were as follows. (1) We identified a “core set” of promoters possessing each epigenetic mark in every surveyed BTSC cell type. However, different BTSC lines showed large numbers of sites differing from the core set for each epigenetic mark. (2) The core set was largely found also in fNSCs, with only a small number of mark locations showing consistent changes from fNSCs to BTSCs. However, for any single BTSC type vs. fNSCs, there were relatively large numbers of differences detected. (3) H3K9me3 showed robust and large differences among cell types in total number of positive promoters and peaks, pointing toward differences in the H3K9me3 producing or maintenance system among these cell types. (4) We detected significant and clear changes in broad modification areas for H3K27me3 and, to a lesser extent, for H3K9me3, implying that the processes creating or maintaining these broad modifications differed in activity among cell types; in one case we verified that this change in a broad modification affected gene expression. (5) We detected large numbers of bivalent promoters. Most bivalent promoters did not display direct overlap of epigenetic mark signals. Rather, the opposing marks seemed to occupy nearby regions of the proximal promoter. (6) There were considerable differences in the sets of promoters bearing bivalent marks in the different cell types and few consistent changes from fNSCs to BTSCs.

A key result of this work was the determination of the “core sets” - a set of promoters for each epigenetic mark that have the epigenetic mark in every BTSC. Because similar cell types show significant similarities in the sets of promoters bearing a given epigenetic mark [25], the existence of a significant core set was not surprising. However, we also detected a large amount of variability among the BTSCs; for each epigenetic mark, the core set comprised well less than half of the total set (universe set) of positive promoters. Given previous gene expression work, this variability among BTSCs is not surprising. Gene expression analyses across glioblastoma multiforme tumors taken *in toto* has shown that there are significant subclasses of glioblastoma that differ in large ways in gene expression patterns [27, 32, 33]. Furthermore, gene expression data from a panel of BTSCs (not including the ones used in this study) indicated significant variability among the BTSCs in gene expression patterns [34]. Finally, preliminary gene expression array data from the BTSCs used in the study (G.Cairncross and S.Weiss, unpublished observations) indicates that the BTSCs used in this study vary significantly in gene expression patterns.

Overall, gene expression studies across different types of BTSCs and neural stem cells demonstrate large sets of genes that are downregulated in one cell type versus another and even in BTSCs as a class vs. neural stem cells (e.g. [34, 35]). A recent analysis [34] concluded that, unlike our previous findings in liver cancer [23], copy number changes explained only a small amount of variation in gene expression between BTSCs and normal neural stem cells and that therefore other mechanisms must be important. Hence, the data in our study, in conjunction with other results, can point toward the potential epigenetic basis of gene repression in these BTSCs. This can be seen most clearly by a brief examination of the recent results of Engström et al. (2012). *TUSC3* is of particular interest because Engström et al. (2012) establish that it is down regulated in glioblastoma cells as compared to neural stem cells and this gene was part of a key set of genes predicting survivability of patients. In bulk glioblastoma tumor samples [36], which comprise both BTSCs and non-BTSCs, this gene is repressed via promoter DNA methylation. However, in our BTSC samples, we find that H3K27me3 in the *TUSC3* promoter may be the major repressor. Similarly, *TES* levels have been described as discriminating normal neural stem cells from BTSCs [34]. This gene is downregulated via promoter DNA methylation in bulk tumor samples [37]. Again, in contrast, we detect strong H3K27me3 signals across BTSC types for this gene’s promoter and little sign of promoter methylation, implying that H3K27me3, not DNA methylation, is probably the repressive mark. These results highlight the importance of studying epigenetic marks directly in BTSCs as opposed to solely tumor samples or other glioblastoma cell lines. Rational targeting of gene expression in BTSCs using drugs aimed at epigenetic processes obviously requires understanding of the epigenetic marks that are affecting gene expression specifically in the context of BTSCs, which may differ from other glioblastoma cell types.

Comparison of the core set for each epigenetic mark with the corresponding set for fNSCs revealed that the core sets were almost entirely found in the corresponding fNSC sets. This indicates that the core set is basically shared not only among the four BTSCs but also fNSCs. However, comparison of the fNSC epigenetic patterns to any single type of BTSC revealed large differences. This may point toward individualized therapeutic targets for patients with a given type of BTSC. Rational drug treatment based on specific properties of a tumor, perhaps even BTSCs derived from an individual tumor, may become a relevant clinical possibility as personalized medicine becomes a larger part of cancer therapy and there is a greater understanding of the overall systems biology of different BTSCs and fNSCs.

Striking and robust differences were found among cell types in the number of promoters bearing the H3K9me3 mark, which points toward different levels of activity of the systems establishing and/or maintaining this mark in these cell types. H3K9me3 appears to have complex actions in gene regulation [16], but clearly plays an important role in neural progenitor cells in producing sustained inactivation of some genes and also plays a large role in some heterochromatic regions [30, 38]. We also noted differences among cell types in large broad regions of modification with H3K9me3. We establish the transcriptional relevance of a difference in an H3K9me3+ broad modification in that it affected transcription of *CACNG7. CACNG7* has been described as being downregulated in BTSCs vs normal neural stem cells [34]. These differences among cell types, in particular among some of the BTSCs and fNSCs, present the intriguing possibility that this H3K9me3 system may be a relevant target for anti-BTSC therapy.

We detected differences among cell types in broad modification regions for H3K27me3 in many of the more lengthy regions that were tiled in this array format. Unfortunately, this array format only covers a portion of the genome, hence, it will be necessary to use other methods, most likely ChIP-seq, to fully establish the prevalence of differences in broad modifications for this mark across different cell types. However, our work provides compelling evidence that broad regions of modification should be a focus of future research, in that regions may play important roles in shaping the biology of these cells and may present therapeutic targets. Targeting the mechanisms that produce these broad regions of epigenetic change represents a potential frontier for epigenetic targeted cancer therapies.

For all five cell types, bivalent promoters, in most cases, had the two opposing epigenetic marks occupying different portions of the proximal promoters, although usually the marks were found directly adjacent to each other. This presents a different picture than that usually associated with bivalent promoters, in which the epigenetic mark signals actually overlap each other [29]. The observation of adjacency has clear mechanistic implications. First, this observation implies that different histone complexes would have each mark. In contrast, for the case of overlapping opposing marks, the traditional interpretation is that both marks are (often) on the same histone tail. In some cases, this has been directly been shown to be so [20, 29]. Our preliminary gene expression results (G. Cairncross and S. Weiss, unpublished observations) indicate that, like the “overlapping” bivalent promoters, these “non-overlapping” bivalent promoters yield very low gene expression, particularly in the case of H3K4me3+/H3K27me3+ bivalent promoters. We also analyzed the case of H3K4me3+/H3K9me3+ bivalent promoters. These bivalent promoters were rarer than H3K4me3+/H3K27me3+ bivalent promoters. A portion of the bivalent promoter set (either K4/K9 or K4/K27) from any single BTSC type was shared with fNSCs but there was only a small set of bivalent promoters that were shared among all BTSCs and fNSCs.

The BTSCs in this study are multipotent [18], as are fNSCs. Because bivalent promoters have been thought important for multipotency, we initially hypothesized that the bivalent promoter set shared among all BTSCs and fNSCs (the common set) would be relatively large in absolute number and as a proportion of all bivalent promoters in these cells. We can rationalize the relatively small common set and relatively large set of bivalent promoters for each individual cell type in several ways. First, it is possible that the cancerous nature of the BTSCs has produced other genomic alterations that affect the bivalent promoter system. Second, bivalent promoters may play important roles in allowing rapid committed cell production but may not be essential for it [28]. Third, there may be several different sets of bivalent promoters that can all support multipotency. Fourth, bivalent promoters may be less important for multipotency than previously thought. Fifth, only a small conserved set of bivalent promoters may be important for multipotency, while most bivalent promoters have other or minor roles. Our common set of bivalent promoters across cell types may include these critical promoters. Sixth, differences in bivalent promoter sets may, in part, produce biases toward production of different types of committed lineage cells. Differentiating among these possibilities will require extensive further investigation.

In general, the spectrum of similarities and differences in sites of epigenetic marks shown in this study suggest different programs of epigenetic change (versus neural stem cells or other cell types of origin) for these different BTSCs. In particular, given the growing evidence for the dynamic nature of epigenetic marks at many sites and the need for active maintenance of these marks at many sites [39], these sets of differences may be actively maintained in each cell type. With this scenario, there may be many points of intervention for possible therapeutic targeting. Although gene ontology analyses of cell type differences revealed some suggestive categories, there were no striking findings of differences. Successful therapeutic intervention may rely on gaining a more detailed, systems biology view of these different cell types. While this may seem a daunting prospect, the rapid advances in high-throughput technologies, particularly in omics fields, coupled with declining costs and higher quality datasets can provide hope for this possibility.

## Conclusions

In total, this work takes significant steps toward understanding the epigenetic marks in these BTSCs on a large genomic scale. We establish a core set of promoters for each epigenetic mark that bear the mark across every BTSC. Our work also points toward mechanisms of repression for genes in these cells, which is of particular interest as BTSC gene expression profiles and patient survival time are correlated [34]. We find substantial differences among cell types in broad modifications and bivalent promoters. These results indicate substantial variation in activity of the production/maintenance systems for H3K9me3 and for broad regions of modification (H3K27me3 or H3K9me3). Interestingly, bivalent promoters often had the two opposing marks immediately adjacent to each other in the proximal promoter as opposed to having direct overlap. The large variability among BTSCs may indicate that the systems biology of these cells may vary greatly and that future epigenetic therapies should be targeted toward subpopulations of patients, or in the extreme, individual patients.

## Methods

### GBM BTSC and fNSC Cell Culture

All glioblastoma multiforme (GBM) BTSC and fNSC neurosphere cell lines were kindly donated by Dr. Sam Weiss and Dr. Greg Cairncross (University of Calgary, Calgary, Alberta, Canada) [18]. The cell types referenced in this manuscript use the same numbering scheme as those previously published [18], e.g. B25 in this manuscript is the same cell line as “BT025” as referenced in [18]. Isolation of fresh tumour samples and the establishment of BTSC and fNSC neurosphere culture have been previously described [18]. Initial, limited, characterization of these cells [18] indicates that B12 cells have amplification of the EGFR locus and that B73 cells possess p53 mutations. All cells were cultured from tumors from male patients, aged 50-70, diagnosed with glioblastoma stage IV (Table S1 of [18]). The GBM BTSCs and fNSC neurospheres were maintained and expanded in serum-free NS media (NeuroCult NS-A media, StemCell Technologies, Cat: 05751) supplemented with EGF (20 ng/mL, Peprotech, Cat: AF-100-15), FGF (20 ng/mL, R&D Systems, Cat: 233-FB) and heparin sulfate (2 μg/mL, StemCell Technologies, Cat: 07980). BTSC and fNSC neurospheres were passaged when the sphere reached an adequate size (200 ~ 400 microns). For cell passaging, the neurospheres were dissociated into single cells by treating with Accumax (Innovative Cell Technologies, #AM105) for 10 min at 37°C and gently triturating using P200 pipettes (Gilson). Cells were then counted and replated. When necessary, cells were fed weekly by adding the supplementary factors listed above (FGF 20 ng/mL, EGF 20 ng/mL, heparin 2 μg/mL).

### Chromatin Immunoprecipitation Assay (ChIP), Methylated DNA Immunoprecipitation Assay (MeDIP), ChIP-chip and MeDIP-chip

Chromatin immunoprecipitation (ChIP) assays were performed as previously described [23] with the following modifications. Briefly, BTSC and fNSC neurospheres were harvested and the spheres were dissociated into a single cell suspension by treating them with Accumax (Innovative Cell Technologies) for 10 min at 37°C followed by triturating with P200 Pipettes. Dissociated BTSC and fNSC cells were cross-linked with 1.0% (final concentration) formaldehyde for 15 min. Nuclear extracts were prepared and sonicated. We followed standard ChIP assay protocols (http://farnham.genomecenter.ucdavis.edu/pdf/FarnhamLabChIP%20Protocol.pdf). Methylated DNA immunoprecipitation (MeDIP) assays used a selective 5-methylcytidine antibody (Eurogentec, BI-MECY-0500; previous used in MeDIP-chip [23, 25]) and genomic DNA was extracted using DNeasy Blood & Tissue Kit (QIAGEN). Extracted genomic DNA was sonicated (Bioruptor Sonicator (Diagenode)) to an average size of 600 bp, denatured at 95°C for 10 min, and quickly chilled on ice prior to ChIP assay using Staphylococcus aureus protein A-positive cells. Antibodies used for the ChIP assays include H3me3K27 (rabbit polyclonal, 5.0 μg, Upstate 07-449; previous used for ChIP-chip work in [23–25]), H3me3K4 (rabbit polyclonal, 6.0 μg, Diagenode PAb-003-050, previously used in ChIP-seq studies [40, 41]), and H3me3K9 (rabbit polyclonal, 3.0 μg, Abcam ab8898, previously used in ChIP-chip studies [24, 25]). The secondary rabbit anti-mouse IgG was purchased from MP Biomedicals (Cat # 55436). Standard PCR reactions using 2 μL of the immunoprecipitated DNA were performed to confirm the ChIP assay, followed by standard electrophoresis procedures (1.5% agarose gels and ethidium bromide visualization). Amplicons, prepared using 50% to 80% of a ChIP sample, were generated using Sigma’s Whole Genome Amplification Kit 2; see previously published ChIP protocol (1) for details. Quality of the amplicons was monitored by PCR of positive and negative control regions. Re-amplification of the amplicons was prepared using Genomeplex WGA Reamplification Kit (WGA3, Sigma). We used the NimbleGen HD2 human promoter microarray design, which focuses on human promoters spanning 7 kb upstream and 3 kb downstream around transcription start sites. The amplicons were sent to NimbleGen Inc (Madison, WI) for application to the promoter microarrays using the standard NimbleGen procedures for these arrays (protocol available at http://www.google.ca/url?sa=t&rct=j&q=&esrc=s&source=web&cd=1&ved=0CB8QFjAA&url=http%3A%2F%2Fwww.nimblegen.com%2Fdownloads%2Fsupport%2F06584098001_NG_Epigenetics_UGuide_v1p0.pdf&ei=LRMCVPnhK5CuogSCl4LwDw&usg=AFQjCNFNqsXPoZOG5n4eP3TOblGpuUfHsw&bvm=bv.74115972,d.cGU). Briefly, 10 ug of Cy5-labeled ChIP DNA and 10 ug of Cy3-labeled input DNA were applied to the arrays. A complete description of this microarray design, including the exact sequence for each spot on the array and the associated data files, is available as part of the GEO platform description for this microarray (http://www.ncbi.nlm.nih.gov/geo/query/acc.cgi?acc=GPL9464).

### Data Analysis

All analyses were with the hg18 genome assembly (see UCSC genome browser: https://genome.ucsc.edu/) and all specified coordinates reference this assembly unless otherwise noted. All microarray data is available via NCBI GEO (GSE60806). We employed a number of different analysis approaches to check the validity of our conclusions. We used our previously developed software [42] and custom software. All quoted values for array amplitudes in this manuscript are in log2 enrichment units, i.e. log2 (ChIP/input DNA). We began with the full set of putative epigenetic sites as determined by the standard NimbleGen analysis; this is the set indicated by the term “generous parameters”. This set was used in all analyses unless otherwise indicated (bivalent promoters were defined differently - see below). To generate these peaks, NimbleGen describes the procedure as follows. The mean and standard deviation of the probe distribution is calculated and a theoretical maximum is calculated as the mean plus six standard deviations. Peaks are then found by searching for at least 4 probes within a 500 bp sliding window with signals above specified cutoff values, ranging from 90% to 15% of the maximum. If a window has less than 4 probes, then 3 probes are searched for and then 2. The full set includes all peaks found by using this procedure. Ratio data were randomized 20 times to calculate the false discovery rate (FDR) for each peak. More details are available in http://www.google.ca/url?sa=t&rct=j&q=&esrc=s&source=web&cd=1&ved=0CB8QFjAA&url=http%3A%2F%2Fwww.nimblegen.com%2Fdownloads%2Fsupport%2F06584098001_NG_Epigenetics_UGuide_v1p0.pdf&ei=LRMCVPnhK5CuogSCl4LwDw&usg=AFQjCNFNqsXPoZOG5n4eP3TOblGpuUfHsw&bvm=bv.74115972,d.cGU. This full set enabled us to determine all possible sites of modification within this microarray design. We then mapped these peaks to proximal promoters using the NimbleGen “5 kb” promoter array design for hg18 (see [24]), which features promoters spanning 3.5 kb upstream and 750 bp downstream. A promoter was considered positive for a modification if one or more NimbleGen peaks overlapped the promoter. We used this promoter-centric analysis for all data analyses except initial quantification of peaks. We favored this approach primarily because epigenetic modifications in proximal promoter regions probably have large effects on transcription. These regions are well represented in this promoter-centric microarray design, allowing us to accurately study this portion of the genome. In addition, because this analysis method focuses on assigning ~4 - 5 kb genomic regions (proximal promoters) to the positive or negative sets as opposed to comparing exact peak locations, it can obviate some subtle noise issues in ChIP-chip work. For example, noise can affect the determination of the “width” and exact location of a given peak, making detailed peak by peak analysis potentially very sensitive to noise. By looking at larger genomic intervals as in proximal promoters, we greatly reduce most issues surrounding detailed analysis of peak widths and associated noise concerns. We produced the “universe” set of promoters for a given epigenetic mark by forming, for each epigenetic mark, the union of positive promoters across BTSC types. In contrast, the core set represents, for each epigenetic mark, the promoters that are positive for that mark in every BTSC type. As an example, if a promoter is positive for H3K4me3 in only B12 cells and not in the other cell types, then it is in the H3K4me3 universe set but not the core set. If a different promoter is positive for H3K4me3 in all four BTSC types, then it is in the H3K4me3 core set and the H3K4me3 universe set. We repeated all these basic analyses (generation of positive promoters for each cell type/epigenetic mark combination, “core” and “universe” set determination) using peaks filtered so that they have FDR < 0.2 (datasets available in Additional file 58). We found that use of this criterion did not lead to significant changes in our overall conclusions (see Additional file 1: Figures S8, S9). For determination of bivalent promoters, we wished to use a more strict criterion because we wanted a set that had strong signals for both epigenetic modifications (either H3K4me3+/H3K27me3+ or H3K4me3+/H3K9me3+). We began by using quantile normalization (across arrays for the same epigenetic mark) and then conservatively smoothing each array dataset using a three point median smooth. Displayed data is generally from this analysis procedure. We next remapped the data to the “5 kb promoters” (as above) and applied maxfour, a methodology used to score promoters for ChIP-chip [43]. In brief, this approach scans each promoter region for the four consecutive probes yielding the largest mean value, which is the “maxfour value” for the promoter. We considered values >1.0 (log2 enrichment, hence >2-fold) as showing enough enrichment to indicate the presence of a peak in the promoter and the promoter is scored as “maxfour positive” for a factor under this condition. Hence, only promoters that were both maxfour-positive and were scored as positive as indicated above by NimbleGen were considered to be in this conservative set. We verified our overall qualitative peak results (e.g. significant variability among BTSCs in sets of promoters bearing a given epigenetic mark) by using other analysis methods. In particular, we used various criteria as in [25] to form a rank analysis of promoters and examine overlap in, for example, the top 2000 or top 4000 promoters as determined by maxfour values. These analyses led to very similar qualitative results as our standard analysis; for example, BTSCs also showed large variability in sets of promoters with a given epigenetic mark according to this approach. For broad modification analyses, we examined the 44 ENCODE regions [44] present in this array design and examined each region for each of the four epigenetic marks by eye. We operationally defined a broad modification as >50 kb in length. Only regions that clearly displayed extended broad peaks that differed among cell types were considered to show a broad modification difference. For quantification of overlap of peaks in bivalent promoters, we wrote custom software. This software only analyzed promoters for which the peak was entirely within the promoter, so the numbers that could be analyzed were a subset of the total. Refinement of this analysis is in process. For gene categorization, we used the DAVID server using standard parameters [26]. This resource not only uses gene ontology categories in the standard analysis, but also uses other classifications (e.g. “SP_PIR_KEYWORDS”); details are available online (http://david.abcc.ncifcrf.gov/) and from [26].

### Isolation of RNA, Preparation of cDNA Library, and qPCR

BTSC and fNSC RNA were prepared using Trizol® Reagent (Invitrogen). No DNase treatement was used. cDNA library was generated using Omniscript RT Kit (QIAGEN) and Oligo(dT)20 primer (Invitrogen) to a volume of 20 uL. Typically 1 uL of this cDNA library was used per qPCR reaction, with the same amount for every well in a given experiment. qPCR was performed in triplicate with GAPDH and ACTB as control genes for normalization using SYBR green as a detector. All primer sets were confirmed to produce a single band product using conventional gel-PCR and a single peak in melting curve analysis, which was performed for every well at the conclusion of the experiment. Experiments were performed using a BioRad CFX-96 qPCR instrument (Bio-Rad Inc., Hercules, CA, USA). Furthermore, all primer sets were designed to produce a product crossing exon-intron-exon boundaries, with the goal of minimizing any effects from genomic DNA contamination. Values are from simple delta-delta Ct analysis [45]. Primers were: CACNG7 (TAAAGAACCAAGCCCACCAC, TCAGCCTCTTCCTCGTGTTC); GAPDH (AAAAGGGTCATCATCTCTGC, GGTGCTAAGCAGTTGGTGGT); ACTB (AAGACCTGTACGCCAACACA, GGAGCAATGATCTTGATCTTCA).

## Electronic supplementary material

Additional file 1: Figure S1-S9: **Supplemental Figures.** (PDF 1 MB)

Additional file 2: **List of promoters and associated genes that have a H3K27me3 peak in all four BTSC types (CORE set).** (XLS 411 KB)

Additional file 3: **List of promoters and associated genes that have a H3K4me3 peak in all four BTSC types (CORE set).** (XLS 950 KB)

Additional file 4: **List of promoters and associated genes that have a H3K9me3 peak in all four BTSC types (CORE set).** (XLS 222 KB)

Additional file 5: **List of promoters and associated genes that have DNA methylation in all four BTSC types (CORE set).** (XLS 684 KB)

Additional file 6: **List of promoters and associated genes that have a H3K27me3 peak in at least one BTSC type (UNIVERSE set).** (XLS 1 MB)

Additional file 7: **List of promoters and associated genes that have a H3K4me3 peak in at least one BTSC type (UNIVERSE set).** (XLS 2 MB)

Additional file 8: **List of promoters and associated genes that have a H3K9me3 peak in at least one BTSC type (UNIVERSE set).** (XLS 1 MB)

Additional file 9: **List of promoters and associated genes that have DNA methylation in at least one BTSC type (UNIVERSE set).** (XLS 2 MB)

Additional file 10: **Output of DAVID gene classification tool for genes that have H3K27me3 in their promoters and associated genes in every BTSC type.** (XLS 802 KB)

Additional file 11: **Output of DAVID gene classification tool for genes that have H3K4me3 in their promoters in every BTSC type.** (XLS 1 MB)

Additional file 12: **Output of DAVID gene classification tool for genes that have H3K9me3 in their promoters in every BTSC type.** (XLS 224 KB)

Additional file 13: **Output of DAVID gene classification tool for genes that have DNA methylation in their promoters in every BTSC type.** (XLS 752 KB)

Additional file 14: **List of promoters and associated genes that are H3K27me3+ in all BTSCs but not in fNSCs.** (XLS 68 KB)

Additional file 15: **List of promoters and associated genes that are H3K4me3+ in all BTSCs but not in fNSCs.** (XLS 46 KB)

Additional file 16: **List of promoters and associated genes that are H3K9me3+ in all BTSCs but not in fNSCs.** (XLS 24 KB)

Additional file 17: **List of promoters and associated genes that have DNA methylation in all BTSCs but not in fNSCs.** (XLS 62 KB)

Additional file 18: **List of promoters and associated genes that are H3K27me3+ in all five examined cell types (4 BTSC types and fNSCs).** (XLS 325 KB)

Additional file 19: **List of promoters and associated genes that are H3K4me3+ in all five examined cell types (4 BTSC types and fNSCs).** (XLS 822 KB)

Additional file 20: **List of promoters and associated genes that are H3K9me3+ in all five examined cell types (4 BTSC types and fNSCs).** (XLS 194 KB)

Additional file 21: **List of promoters and associated genes that have DNA methylation in all five examined cell types (4 BTSC types and fNSCs).** (XLS 575 KB)

Additional file 22: **List of promoters and associated genes that are H3K27me3+ in fNSCs but not any type of BTSC (unique to fNSCs).** (XLS 104 KB)

Additional file 23: **List of promoters and associated genes that are H3K4me3+ in fNSCs but not any type of BTSC (unique to fNSCs).** (XLS 96 KB)

Additional file 24: **List of promoters and associated genes that are H3K9me3+ in fNSCs but not any type of BTSC (unique to fNSCs).** (XLS 182 KB)

Additional file 25: **List of promoters and associated genes that have DNA methylation in fNSCs but not any type of BTSC (unique to fNSCs).** (XLS 122 KB)

Additional file 26: **Output of DAVID gene classification tool for genes that have H3K27me3 in their promoters in every BTSC type but not in fNSCs.** (XLS 286 KB)

Additional file 27: **Output of DAVID gene classification tool for genes that have H3K27me3 in their promoters in fNSCs but not any BTSC type (unique to fNSCs).** (XLS 479 KB)

Additional file 28: **Output of DAVID gene classification tool for genes that have H3K4me3 in their promoters in every BTSC type but not in fNSCs.** (XLS 194 KB)

Additional file 29: **Output of DAVID gene classification tool for genes that have H3K4me3 in their promoters in fNSCs but not any BTSC type (unique to fNSCs).** (XLS 462 KB)

Additional file 30: **Output of DAVID gene classification tool for genes that have H3K9me3 in their promoters in every BTSC type but not in fNSCs.** (XLS 42 KB)

Additional file 31: **Output of DAVID gene classification tool for genes that have H3K9me3 in their promoters in fNSCs but not any BTSC type (unique to fNSCs).** (XLS 866 KB)

Additional file 32: **Output of DAVID gene classification tool for genes that have methylated DNA in their promoters in every BTSC type but not in fNSCs.** (XLS 319 KB)

Additional file 33: **Output of DAVID gene classification tool for genes that have methylated DNA in their promoters in fNSCs but not any BTSC type (unique to fNSCs).** (XLS 587 KB)

Additional file 34: **Listing of broad regions for each modification that are differentially present among cell types.** (XLS 28 KB)

Additional file 35: **List of bivalent promoters and associated genes (H3K4me3+/H3K27me3+) in B12 cells.** (XLS 150 KB)

Additional file 36: **List of bivalent promoters and associated genes (H3K4me3+/H3K9me3+) in B12 cells.** (XLS 100 KB)

Additional file 37: **List of bivalent promoters and associated genes (H3K4me3+/H3K27me3+) in B25 cells.** (XLS 124 KB)

Additional file 38: **List of bivalent promoters and associated genes (H3K4me3+/H3K9me3+) in B25 cells.** (XLS 64 KB)

Additional file 39: **List of bivalent promoters and associated genes (H3K4me3+/H3K27me3+) in B48 cells.** (XLS 168 KB)

Additional file 40: **List of bivalent promoters and associated genes (H3K4me3+/H3K9me3+) in B48 cells.** (XLS 71 KB)

Additional file 41: **List of bivalent promoters and associated genes (H3K4me3+/H3K27me3+) in B73 cells.** (XLS 218 KB)

Additional file 42: **List of bivalent promoters and associated genes (H3K4me3+/H3K9me3+) in B73 cells.** (XLS 84 KB)

Additional file 43: **List of bivalent promoters and associated genes (H3K4me3+/H3K27me3+) in fNSCs.** (XLS 289 KB)

Additional file 44: **List of bivalent promoters and associated genes (H3K4me3+/H3K9me3+) in fNSCs.** (XLS 112 KB)

Additional file 45: **Output of DAVID gene classification tool for genes that are bivalent (H3K4me3+/H3K27me3+) in their promoters in fNSCs and are only H3K27me3+ in all BTSC types.** (XLS 104 KB)

Additional file 46: **Output of DAVID gene classification tool for genes that are bivalent (H3K4me3+/H3K27me3+) in their promoters in fNSCs and in all BTSC types.** (XLS 40 KB)

Additional file 47: **Output of DAVID gene classification tool for genes that are bivalent (H3K4me3+/H3K27me3+) in their promoters in fNSCs and are only H3K4me3+ in all BTSC types.** (XLS 48 KB)

Additional file 48: **Output of DAVID gene classification tool for genes that are bivalent (H3K4me3+/H3K27me3+) in their promoters in fNSCs and are H3K4me3-/H3K27me3- in all BTSC types.** (XLS 226 KB)

Additional file 49: **Output of DAVID gene classification tool for genes that are bivalent (H3K4me3+/H3K9me3+) in their promoters in fNSCs and in all BTSC types.** (XLS 38 KB)

Additional file 50: **Output of DAVID gene classification tool for genes that are bivalent (H3K4me3+/H3K9me3+) in their promoters in fNSCs and are only H3K4me3+ in all BTSC types.** (XLS 52 KB)

Additional file 51: **Output of DAVID gene classification tool for genes that are bivalent (H3K4me3+/H3K9me3+) in their promoters in fNSCs and are only H3K9me3+ in all BTSC types.** (XLS 34 KB)

Additional file 52: **List of bivalent promoters and associated genes (H3K4me3+/H3K27me3+) in all five cell types.** (XLS 30 KB)

Additional file 53: **List of bivalent promoters and associated genes (H3K4me3+/H3K9me3+) in all five cell types.** (XLS 26 KB)

Additional file 54: **List of bivalent promoters and associated genes (H3K4me3+/H3K27me3+) in fNSCs and not bivalent in any BTSC.** (XLS 18 KB)

Additional file 55: **List of bivalent bivalent promoters and associated genes (H3K4me3+/H3K27me3+) in all BTSCs and not fNSCs.** (XLS 16 KB)

Additional file 56: **List of bivalent promoters and associated genes (H3K4me3+/H3K9me3+) in fNSCs and not bivalent in any BTSC.** (XLS 18 KB)

Additional file 57: **List of bivalent promoters and associated genes (H3K4me3+/H3K9me3+) in all BTSCs and not fNSCs.** (XLS 16 KB)

Additional file 58: **FDR < 0.2 datasets of positive promoters and associated genes by cell type (5 cell types × 4 epigenetic marks) and core and universe sets for BTSCs (28 worksheets).** The initial worksheet contains contents and explanation. (XLS 10 MB)
