# Supplementary material for: Differences among brain tumor stem cell types and fetal neural stem cells in focal regions of histone modifications and DNA methylation, broad regions of modifications, and bivalent promoters
Source: BMC Genomics. 2014 Aug 27;15(1):724. doi: 10.1186/1471-2164-15-724 (PMC4155105; doi:10.1186/1471-2164-15-724)
Supplement: Supplementary file 1 — Additional file 1: Figure S1-S9: Supplemental Figures. (PDF 1 MB) [file 12864_2014_6396_MOESM1_ESM.pdf]

Figure Legends

Figure S1. Validation of H3K4me3+ microarray data. A. Microarray data from TXN promoter (chr9) for H3K4me3 ChIP-chip experiments across five cell types. Note great similarity in waveforms across cell types. B. B73 cell data across four epigenetic modifications for TXN promoter. Note only significant peak is in H3K4me3 data. C. ENCODE data from nine cell types for H3K4me3 ChIP-seq experiments. Note similarity in range and shape of this data to ChIP-chip data in A. Left axes are log2 enrichment for A, B and counts in C. Data downloaded from UCSC browser July 1, 2014.

Figure S2. Validation of H3K27me3 microarray data. A. Microarray data from HOXB cluster (chr17) for H3K27me3 ChIP-chip experiments across five cell types. Note great similarity in waveforms across cell types. B. B73 cell data across four epigenetic modifications for in this region. Note only significant peak is in H3K27me3 data. C. ENCODE data from H1-hESC (H1 human embryonic stem cells) for H3K27me3 ChIP-seq experiments. Note similarity in range and shape of this data to ChIP-chip data in A. Left axes are log2 enrichment for A, B and counts in C. Data downloaded from UCSC browser July 1, 2014.

Figure S3. Validation of H3K9me3 microarray data. A. Microarray data from ZNF260 promoter (chr19) for H3K9me3 ChIP-chip experiments across five cell types. Note great similarity in waveforms across cell types. B. B73 cell data across four epigenetic modifications for in this region. Note H3K9me3 enrichment surrounds peak of H3K4me3 enrichment. C. ENCODE data from HepG2 cells for H3K9me3 ChIP-seq experiments. Note this data is hg19 coordinates while A and B are in hg18 coordinates. Note similarity in range and shape of this data to ChIP-chip data in B. In particular, H3K4me3 signal relationship to H3K9me3 is very similar between technologies. Left axes are log2 enrichment for A, B and counts in C. Data downloaded from UCSC browser July 1, 2014.

Figure S4. Validation of H3K9me3 microarray data across a zinc-finger gene region. Zinc finger-rich regions on chromosome 19 are well known sites of H3K9me3 signals [24]. A. Microarray data from a genomic region rich in zinc finger genes (chr19) for H3K9me3 ChIP-chip experiments across five cell types. Note strong similarities in waveforms across cell types. B. B73 cell data across four epigenetic modifications for in this region. Note H3K9me3 enrichment and H3K4me3 enrichment. C. ENCODE data from HepG2 cells for H3K4me3 ChIP-seq experiments for a portion of this region; this data shows that H3K4me3 signals are expected along with the previously described H3K9me3 peaks. Left axes are log2 enrichment for A, B and counts in C. Data downloaded from UCSC browser July 1, 2014.

Figure S5. Validation of MeDIP-chip data. A. Microarray data from CDX1 gene (chr5) for MeDIP-chip experiments across five cell types. Note great similarity in waveforms across cell types. B. B73 cell data across four epigenetic modifications for this region. Note only significant peak is in DNA methylation data. Axes are lower range because MeDIP signal intensity is less overall than histone ChIP-chip experiments. C. ENCODE data from gliobastoma cell line U87 showing sites of DNA methylation from reduced representation bisulfite sequencing (upper U87 track) and Illumina Infinium Human Methylation 450 Bead Array platform (lower U87 track). Coloring indicates strong signals in both cases. Note hg19 coordinates while MeDIP-chip is hg18

coordinates. Note methylation is in same region as MeDIP signal. Left axes are log2 enrichment for A, B and counts in C. Data downloaded from UCSC browser July 1, 2014.

Figure S6. Global analysis of correlations between microarray datasets using promoter enrichment. For every promoter, the maxfour value (the maximum mean value of four consecutive probes) was calculated. A single point represents a single promoter - the values are the maxfour values for each plotted dataset. A. H3K27me3 microarray data from fNSCs and B73 cells displays many correlated promoters. B. H3K27me3 and H3K9me3 are mostly mutually exclusive. Compare to [24]. C. H3K4me3 and H3K27me3 are mostly mutually exclusive.

Figure S7. Analysis of universe sets for presence of outlier cell types. A. Categorization of each positive promoter in the universe set by whether it occurs in one, two, three, or all four assayed BTSC types (denoted by “All”). Pie chart representation of data. This data is comparable to Figure 2, except that the promoters outside of the CORE set (indicated by “All” here) are further divided into those that occurred in a single type of BTSC, two types, or three types. B. Analysis of contribution of unique positive promoters for each cell type/epigenetic mark combination to total universe of positive promoters. If a single cell type were an outlier possessing little similarity to the others, then the “One” category in A would be large and positive promoters unique to the cell type would be a significant fraction of the total universe set in B. Note that the only significant outlier cell type appears to be B12 for H3K9me3+ promoters (~23% of universe set are promoters that are H3K9me3+ in only B12 cells). However, removal of this cell type from analyses did not significantly affect qualitative conclusions (data not shown).

Figure S8. Number of peak-containing promoters for each cell type and epigenetic mark using relatively stringent peak-finding criteria. Nimblegen peak files (see Methods) were filtered for predicted peaks with FDR < 0.2. These peaks were then mapped to the “5 kb” promoter design as detailed in METHODS. If a promoter overlapped one or more peaks, the promoter was scored as positive. For H3K9me3, these results qualitatively accord with results based on generous peak finding parameters (see Figure 1). The same two cell types (B25, B48) demonstrate much lower numbers of H3K9me3+ promoters than the other types (B12, B73, fNSC) using either these stringent criteria or those of Figure 1.

Figure S9. Analysis of fNSC and BTSC specific positive promoters for promoters with high confidence peaks (FDR < 0.2). This figure is directly comparable to Figure 3 in the main text, which used the full set of peaks (see Methods). Note that many values only shifted by a small amount. All datasets that were used to compute these comparisons are available in Additional File 58.

Figure S1

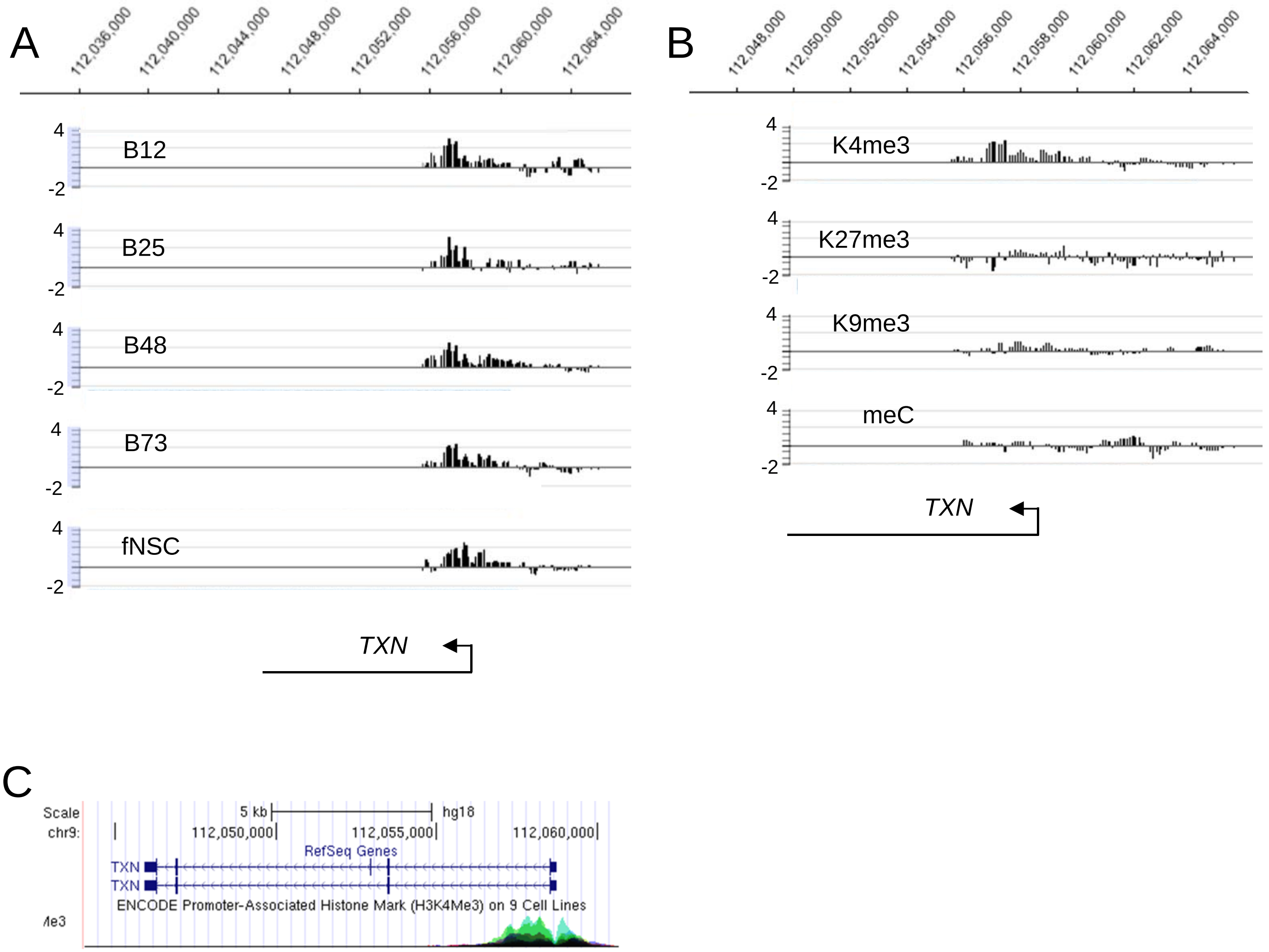
A
112,036,000
112,040,000
112,044,000
112,048,000
112,052,000
112,056,000
112,060,000
112,064,000
B12
B25
B48
B73
fNSC
4
-2
TXN
B
112,048,000
112,050,000
112,052,000
112,054,000
112,056,000
112,058,000
112,060,000
112,062,000
112,064,000
K4me3
K27me3
K9me3
meC
TXN
C
Scale
5 kb
hg18
chr9:
112,050,000
112,055,000
112,060,000
RefSeq Genes
TXN
TXN
ENCODE Promoter-Associated Histone Mark (H3K4Me3) on 9 Cell Lines

Figure S2

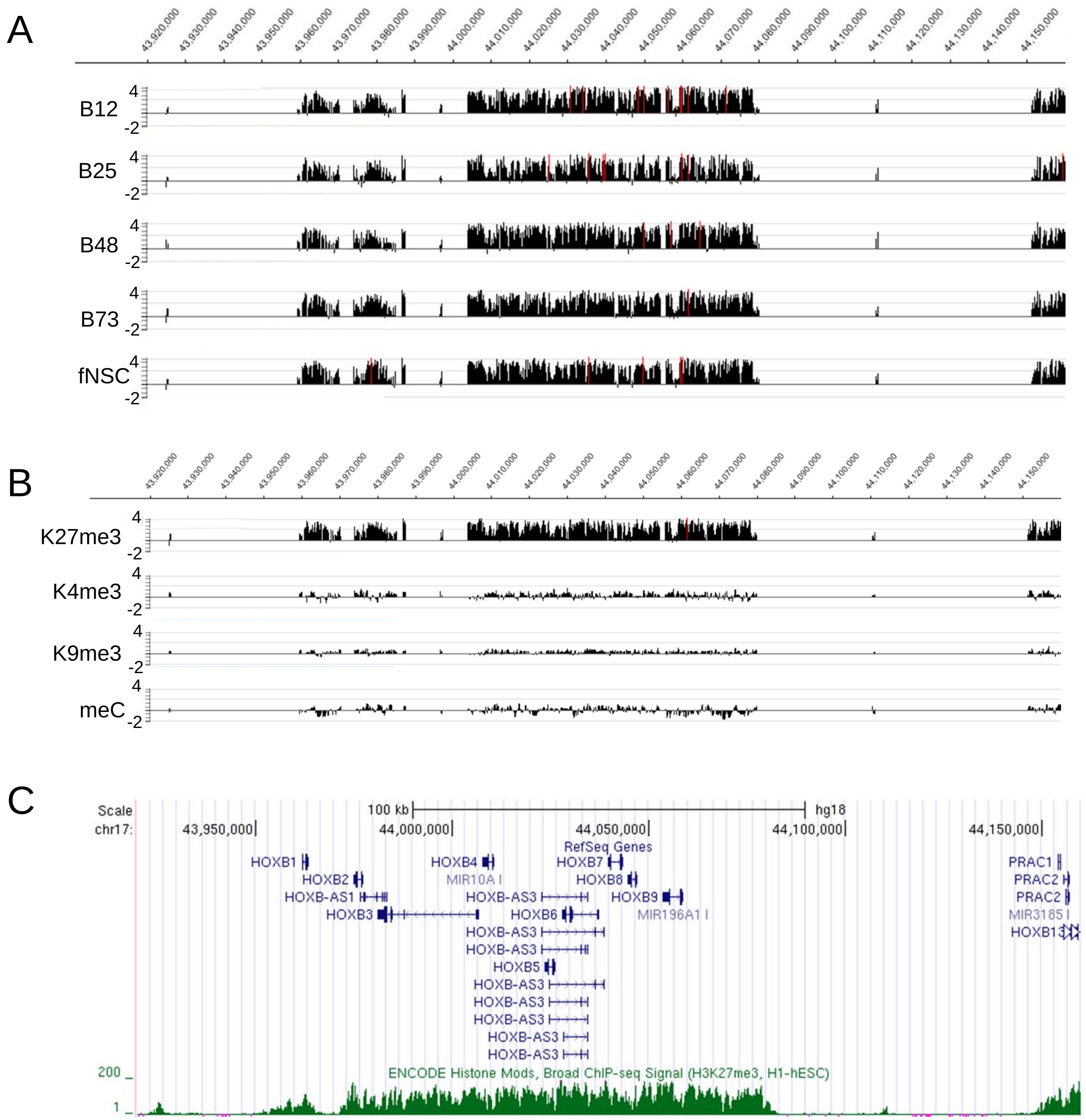

A
43,920,000
43,930,000
43,940,000
43,950,000
43,960,000
43,970,000
43,980,000
43,990,000
44,000,000
44,010,000
44,020,000
44,030,000
44,040,000
44,050,000
44,060,000
44,070,000
44,080,000
44,090,000
44,100,000
44,110,000
44,120,000
44,130,000
44,140,000
44,150,000
B12
B25
B48
B73
fNSC
4
-2
B
K27me3
K4me3
K9me3
meC
C
Scale
100 kb
hg18
chr17:
43,950,000
44,000,000
44,050,000
44,100,000
44,150,000
RefSeq Genes
HOXB1
HOXB2
HOXB-AS1
HOXB3
HOXB4
MIR10A
HOXB-AS3
HOXB7
HOXB8
HOXB9
HOXB6
MIR196A1
HOXB5
PRAC1
PRAC2
MIR3185
HOXB13
200
1
ENCODE Histone Mods, Broad ChIP-seq Signal (H3K27me3, H1-hESC)

Figure S3

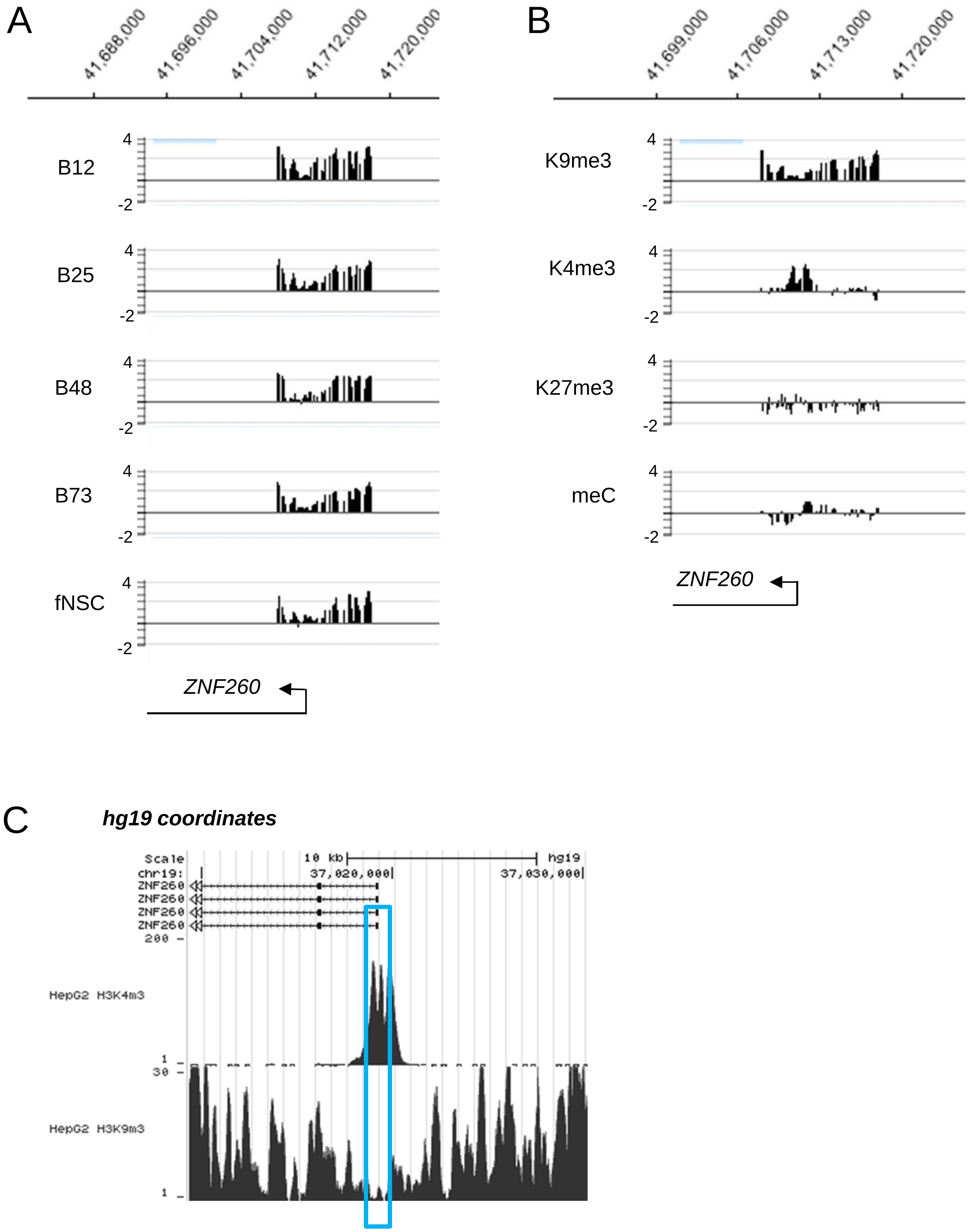

A
41,688,000
41,696,000
41,704,000
41,712,000
41,720,000
B12
B25
B48
B73
fNSC
4
-2
ZNF260
B
41,699,000
41,706,000
41,713,000
41,720,000
K9me3
K4me3
K27me3
meC
ZNF260
C
hg19 coordinates
Scale
10 kb
hg19
chr19:
37,020,000
37,030,000
ZNF260
200
HepG2 H3K4m3
1
30
HepG2 H3K9m3
1

Figure S4

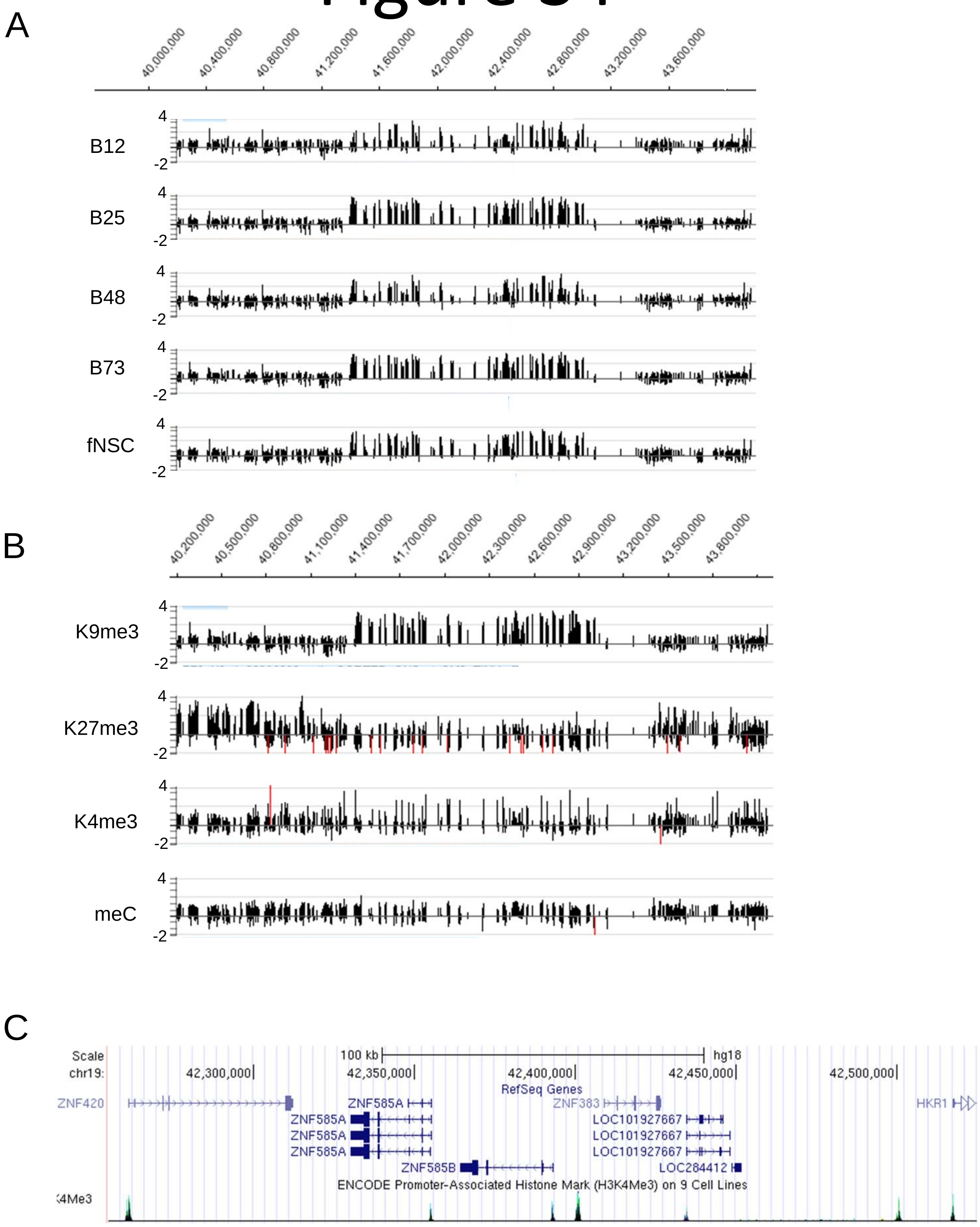
A
40,000,000
40,400,000
40,800,000
41,200,000
41,600,000
42,000,000
42,400,000
42,800,000
43,200,000
43,600,000
B12
B25
B48
B73
fNSC
4
-2
B
40,200,000
40,500,000
40,800,000
41,100,000
41,400,000
41,700,000
42,000,000
42,300,000
42,600,000
42,900,000
43,200,000
43,500,000
43,800,000
K9me3
K27me3
K4me3
meC
C
Scale
chr19:
100 kb
hg18
42,300,000
42,350,000
42,400,000
42,450,000
42,500,000
RefSeq Genes
ZNF420
ZNF585A
ZNF585B
ZNF383
LOC101927667
LOC284412
HKR1
ENCODE Promoter-Associated Histone Mark (H3K4Me3) on 9 Cell Lines

Figure S5

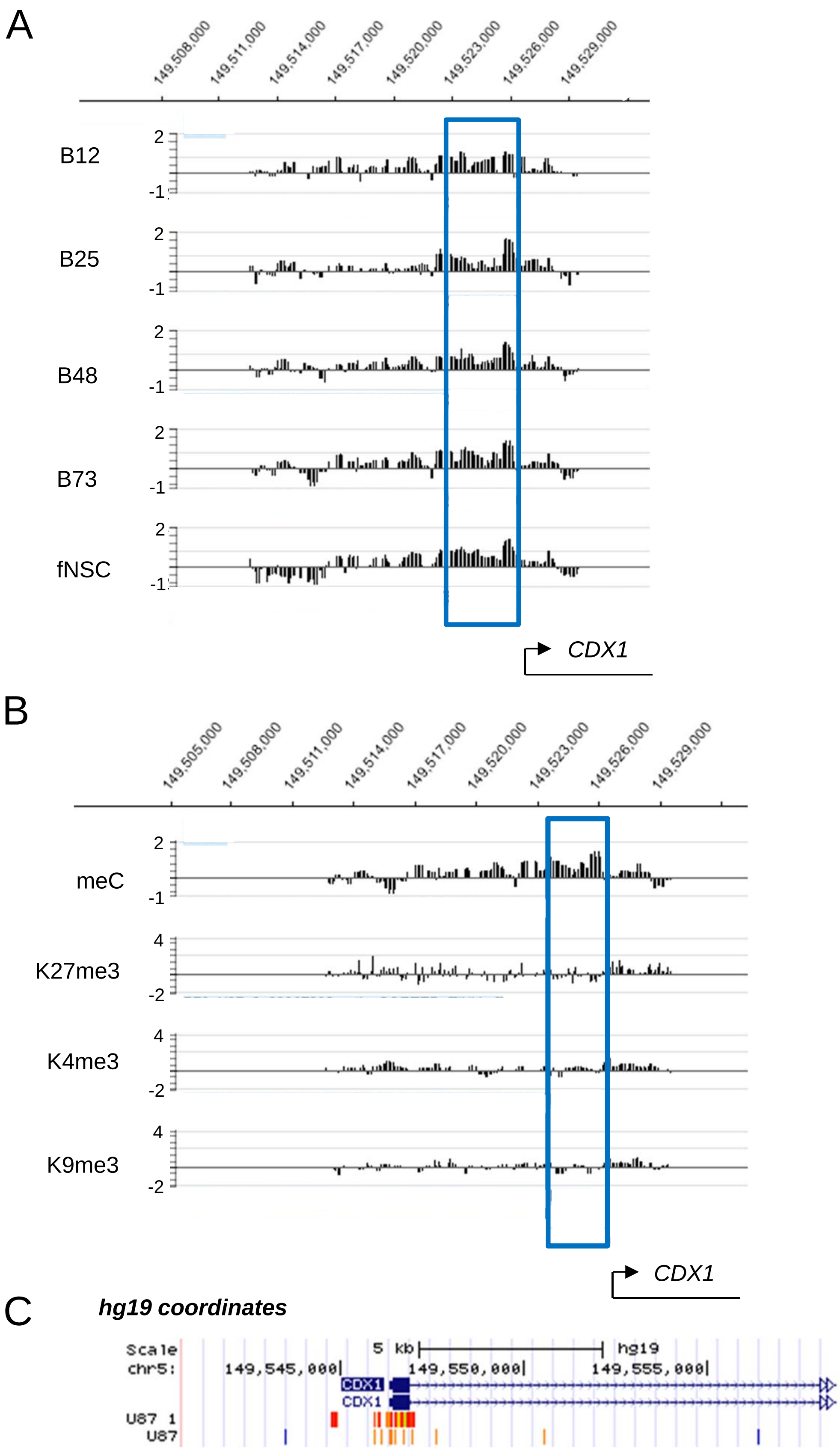
A
149,508,000
149,511,000
149,514,000
149,517,000
149,520,000
149,523,000
149,526,000
149,529,000
B12
B25
B48
B73
fNSC
2
-1
CDX1
B
149,505,000
149,508,000
149,511,000
149,514,000
149,517,000
149,520,000
149,523,000
149,526,000
149,529,000
meC
K27me3
K4me3
K9me3
4
-2
CDX1
C
hg19 coordinates
Scale
chr5:
5 kb
hg19
149,545,000
149,550,000
149,555,000
CDX1
CDX1
U87 1
U87

Figure S6

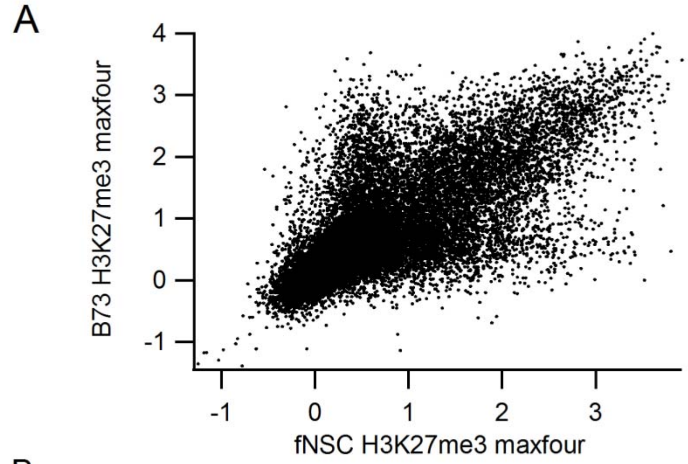

A
4
3
2
1
0
-1
B73 H3K27me3 maxfour
-1
0
1
2
3
fNSC H3K27me3 maxfour

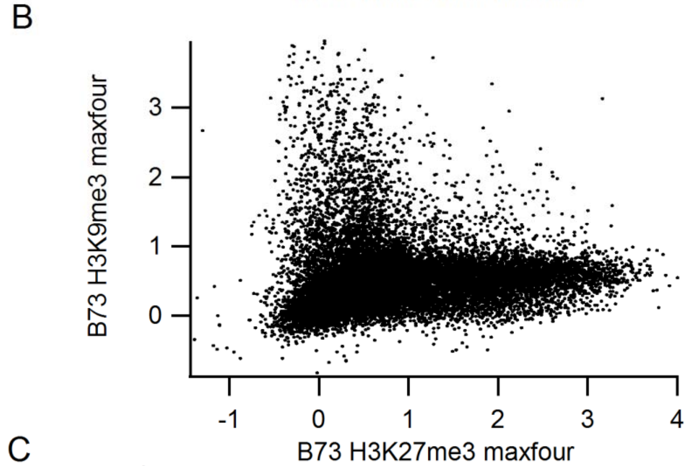

B
3
2
1
0
B73 H3K9me3 maxfour
-1
0
1
2
3
4
B73 H3K27me3 maxfour

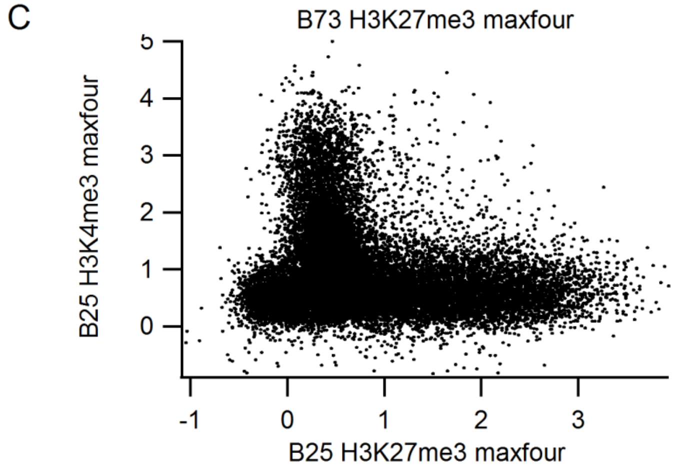

C
5
4
3
2
1
0
B25 H3K4me3 maxfour
-1
0
1
2
3
B25 H3K27me3 maxfour

Figure S7

A

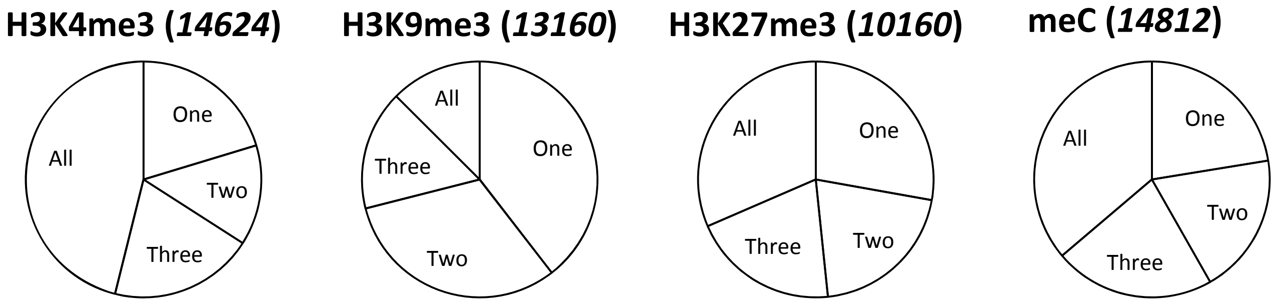

B

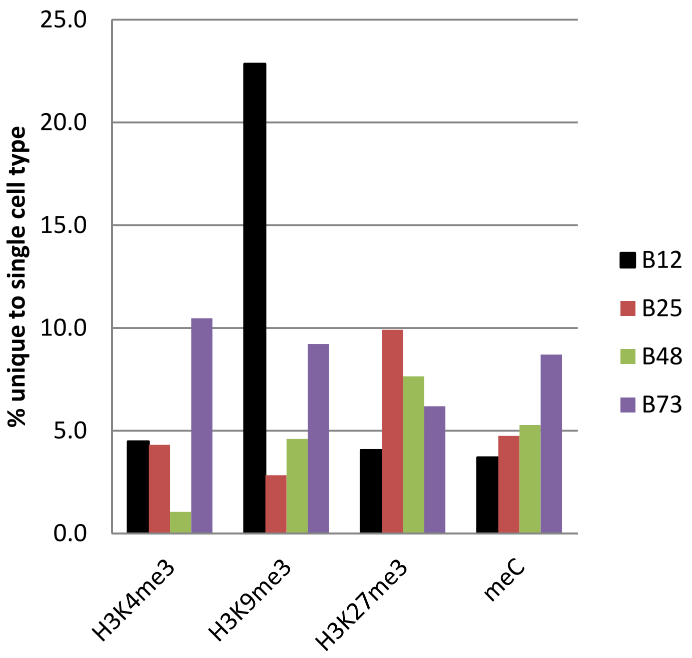

Figure S7. Analysis of universe sets for presence of outlier cell types. A. Categorization of each positive promoter in the universe set by whether it occurs in one, two, three, or all four assayed BTSC types (denoted by “All”). Pie chart representation of data. This data is comparable to Figure 2, except that the promoters outside of the CORE set (indicated by “All” here) are further divided into those that occurred in a single type of BTSC, two types, or three types. B. Analysis of contribution of unique positive promoters for each cell type/epigenetic mark combination to total universe of positive promoters. If a single cell type were an outlier possessing little similarity to the others, then the “One” category in A would be large and positive promoters unique to the cell type would be a significant fraction of the total universe set in B. Note that the only significant outlier cell type appears to be B12 for H3K9me3+ promoters (~23% of universe set are promoters that are H3K9me3+ in only B12 cells). However, removal of this cell type from analyses did not significantly affect qualitative conclusions (data not shown).

Figure S8

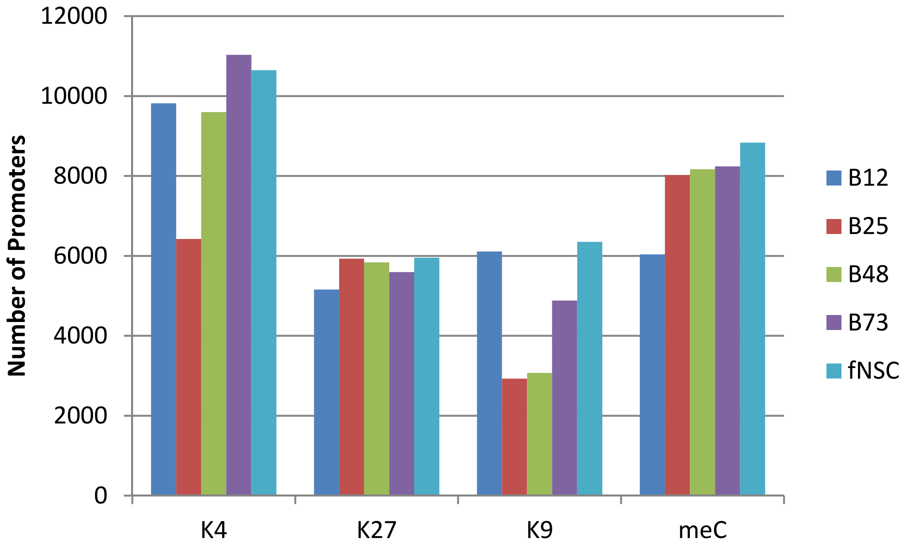

Figure S8. Number of peak-containing promoters for each cell type and epigenetic mark using relatively stringent peak-finding criteria. Nimblegen peak files (see METHODS) were filtered for predicted peaks with FDR<=0.2. These peaks were then mapped to the “5 kb” promoter design as detailed in METHODS. If a promoter overlapped one or more peaks, the promoter was scored as positive. For H3K9me3, these results qualitatively accord with results based on generous peak finding parameters (see Figure 1). The same two cell types (B25, B48) demonstrate much lower numbers of H3K9me3+ promoters than the other types (B12, B73, fNSC) using either these stringent criteria or those of Figure 1.

Figure S9

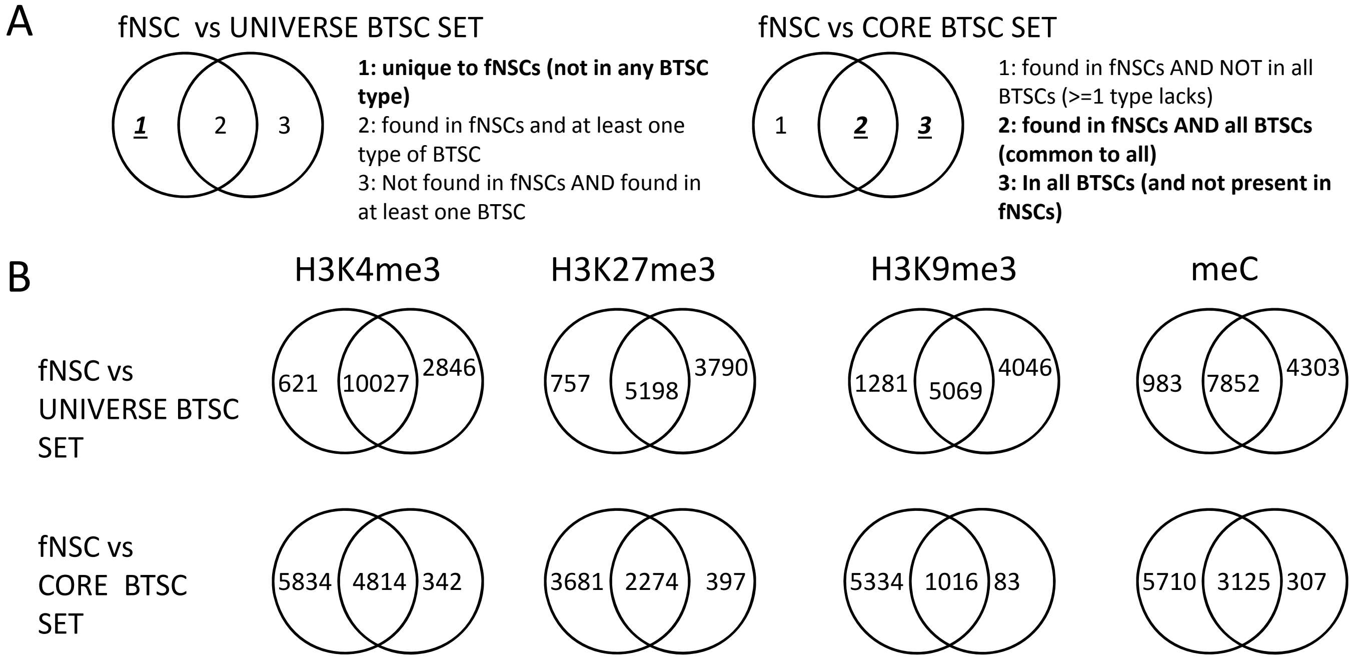

A
fNSC vs UNIVERSE BTSC SET
1 2 3
1: unique to fNSCs (not in any BTSC type)
2: found in fNSCs and at least one type of BTSC
3: Not found in fNSCs AND found in at least one BTSC
fNSC vs CORE BTSC SET
1 2 3
1: found in fNSCs AND NOT in all BTSCs (>=1 type lacks)
2: found in fNSCs AND all BTSCs (common to all)
3: In all BTSCs (and not present in fNSCs)
B
H3K4me3
H3K27me3
H3K9me3
meC
fNSC vs UNIVERSE BTSC SET
621 10027 2846
757 5198 3790
1281 5069 4046
983 7852 4303
fNSC vs CORE BTSC SET
5834 4814 342
3681 2274 397
5334 1016 83
5710 3125 307
